# The Core of the Matter—Importance of Identification Method and Biological Replication for Benthic Marine Monitoring

**DOI:** 10.1002/ece3.70556

**Published:** 2024-11-14

**Authors:** Mads Reinholdt Jensen, Sune Agersnap, Eva Egelyng Sigsgaard, Marcelo de Paula Ávila, Henrik Glenner, Mary S. Wisz, Philip Francis Thomsen

**Affiliations:** ^1^ Department of Biology Aarhus University Aarhus C Denmark; ^2^ Norwegian College of Fishery Science UiT—The Arctic University of Norway Tromsø Norway; ^3^ Center for Sustainable Landscapes under Global Change (SustainScapes) Aarhus University Aarhus C Denmark; ^4^ Department of Biological Sciences University of Bergen Bergen Norway; ^5^ Center of Macroecology and Climate, GLOBE University of Copenhagen Copenhagen Denmark; ^6^ Ocean Sustainability, Governance and Management World Maritime University Malmö Sweden

**Keywords:** benthic monitoring, benthic–pelagic coupling, biomonitoring, community assessment, ecological status, Kattegat

## Abstract

Benthic macrofauna are important and widely used biological indicators of marine ecosystems as they have limited mobility and therefore integrate the effects of local environmental stressors over time. Recently, environmental DNA (eDNA) analysis has provided a potentially more resource‐efficient approach for benthic biomonitoring than traditional morphology‐based methods. Several studies have compared eDNA with morphology‐based monitoring, but few have compared the two approaches using the exact same sediment cores. In addition, the meiofauna and pelagic organisms obtained as ‘bycatch’ using eDNA have largely been disregarded from comparisons. Here, we address these shortcomings through comparative invertebrate analyses of six sediment sample replicates from each of four stations in Denmark, using eDNA metabarcoding and morphological identification. Our results revealed large variation between the six replicates for both methods and little overlap in taxon compositions between methods. While the morphological dataset was dominated by molluscs and annelids, the eDNA dataset was dominated by arthropods and annelids. Using community composition data, we found that sampling stations could be distinguished both with eDNA and morphology. Finally, we inferred expected total richness from extrapolated accumulation curves of detected taxa from each method. This indicated that eDNA metabarcoding requires less replication than morphology for maximum coverage of diversity to be reached. However, both methods required high levels of replication, and our results on taxonomic composition add to the evidence that morphological and eDNA‐based methods should preferably be used as complimentary tools for marine bioassessment.

## Graphical abstract

Benthic macrofauna are important and widely used biological indicators of marine ecosystems as they have limited mobility and therefore integrate the effects of local environmental stressors over time. We here compare environmental DNA metabarcoding obtained from sediments with conventional morphological identification of benthic macrofauna. We identify that high levels of biological replication are needed for both methods to reach taxon saturation and that the two methods should be used in concert as complementary tools for marine biomonitoring.

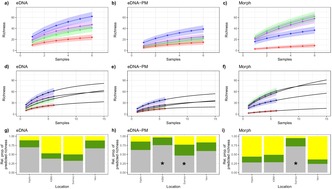

## 1 Introduction

Monitoring of marine benthic communities plays a fundamental role in assessing the ecological status of marine ecosystems, and is included in all governmental regulations that seek to achieve environmental objectives in the oceans, such as the Marine Strategy Framework Directive (MSFD, 2008/56/EC) (Aylagas et al. 2018). Marine benthic communities are both locally and globally important for key biological processes, such as nutrient cycling and carbon decomposition, thus affecting multiple trophic levels and creating a strong benthic–pelagic coupling in the marine environment (Hauer et al. 2018; Kritzer et al. 2016). Together with the limited mobility of most benthic organisms, this makes them useful as biological indicators for marine ecosystems, as they integrate the effects of local environmental stressors over time and across the benthic and pelagic environments (Crespo and Pardal 2020).

In Denmark, the first marine monitoring programme was initiated in 1988 and is part of the ‘National Program for Surveillance of the Aquatic Environment and Nature’ (NOVANA) (Jensen et al. 2017). It consists of annual monitoring of the benthic macrofaunal communities (> 1 mm) in Danish waters using sediment core sampling (Hansen and Høgslund 2021), and includes 72 sampling stations. Each station is monitored for species composition and abundance at least once or twice in every 5‐year period of the programme, and certain stations are monitored every year. Currently, the monitoring effort is based on visual identification, counting and weighing of specimens, which is labour intensive, expensive and requires specialised taxonomic experts (Carugati et al. 2015).

Hence, there is a need for effective and affordable assessment tools for monitoring benthic biodiversity (Heiskanen et al. 2016), and the development of high‐throughput sequencing techniques within the last decade has made metabarcoding of environmental DNA (eDNA) an appropriate candidate for such a tool (Kelly et al. 2016; Taberlet et al. 2012; Thomsen and Willerslev 2015). Environmental DNA metabarcoding has already been demonstrated as a useful approach to monitor marine biodiversity of both pelagic (Adams et al. 2023; Agersnap et al. 2022; Berry et al. 2019; Jensen et al. 2022) and benthic communities (Cahill et al. 2018; Lanzén et al. 2021; Lejzerowicz et al. 2015), but is not yet widely applied in marine management.

A large impediment to implementation of eDNA analyses into national monitoring efforts is that monitoring results need to be comparable from year to year, which is why the same approach has been used for decades. Replacing or supplementing the current approach with eDNA metabarcoding will therefore require thorough benchmarking against the former (Wangensteen et al. 2018). Another key impediment to implementation of DNA‐based techniques into actionable marine environmental management is the lack of standardised protocols to enable comparisons of spatiotemporal biological variance, as well as the lack of translational dialogues among molecular ecologists and stakeholders (Aylagas et al. 2020; Thomsen, Jensen, and Sigsgaard 2024).

Initial benthic metabarcoding studies used bulk samples of invertebrates rather than eDNA samples, which allowed for very direct comparisons with traditional methods (e.g., Aylagas et al. 2018; Cahill et al. 2018; Leray and Knowlton 2015; Lobo et al. 2017). For example, Cahill et al. (2018) found that bulk metabarcoding and morphological identification provided complementary information, and that similar biogeographical patterns could be observed across the two methods. However, several studies have now compared eDNA metabarcoding to conventional morphological surveys for benthic monitoring (e.g., He et al. 2021; Keck et al. 2022; Lanzén et al. 2021; Schenk, Kleinbölting, and Traunspurger 2020). Lanzén et al. (2021) showed that benthic community composition inferred from eDNA performed similarly to morphology‐based methods for monitoring responses to oil drilling activities in Norway. A recent meta‐study by Keck et al. (2022) also found that eDNA metabarcoding provided complementary information regarding the biodiversity and taxonomic composition of benthic communities compared to conventional morphological methods.

An important caveat in previous comparisons of eDNA‐based and morphological identifications is that the sediment grabs/cores were rarely the exact same across the two methods, thus making direct comparisons limited. Furthermore, eDNA input from pelagic or meiofaunal organisms could potentially provide additional resolution when assessing ecological status (i.e., through presence/absence or semiquantitatively with read proportions). However, these organisms are often disregarded from comparisons, as they are not included in the morphology‐based assessment. Here, we address these shortcomings by comparing biomonitoring results obtained from traditional morphological identification and eDNA metabarcoding of the same 24 sediment cores from four ‘NOVANA’ sampling stations in Denmark.

## 2 Methods

### 2.1 Study Area and Sampling

**FIGURE 1:**
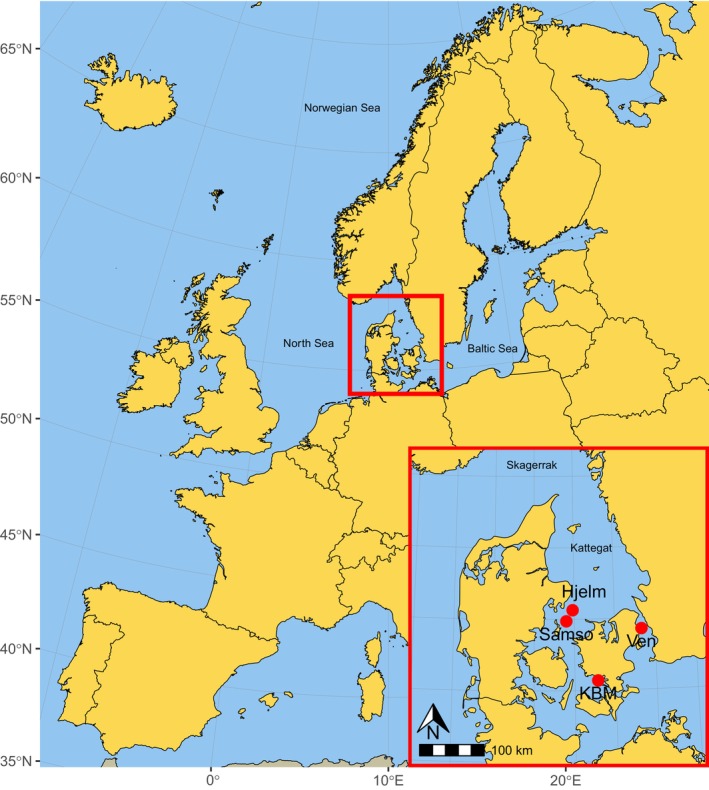
Map of Denmark with red circles indicating the four sampling stations: Hjelm, Samso, Ven and Karrebaeksminde (KBM).

Sampling was carried out in collaboration with the Danish Environmental Protection Agency (EPA) onboard three different survey vessels during the annual benthic macrofaunal monitoring survey carried out in March and April 2019. The complete survey includes sampling of ~40 different stations over a 2‐month period and collection of 10–42 individual sediment cores from each station. Given the large variation in species communities found from core to core within a station, all sampling stations have higher sampling efforts in certain years to ensure a better resolution of the variation. Each sediment core is directly washed through a 1 mm mesh sieve in the field, and the residual macrofaunal species are then preserved in 96% ethanol. In the laboratory, the macrofaunal specimens are identified morphologically to the lowest taxonomic level possible, counted and weighed, as described in detail by Hansen and Josefson (2014). The current project covered four survey stations in the inner Danish waters (Baltic Sea), namely, Hjelm, Karrebaeksminde (KBM), Samso and Ven (Figure 1, Table S1). These four stations were chosen as they are known from previous monitoring data to exhibit clear differences in species diversity. The areal extent of these sampling stations varies from < 1 to > 10 km^2^ (Samso: 0.003, Hjelm: 0.02, Ven: 0.3 and KBM: 13.8 km^2^).

At these four stations, an additional six individual sediment cores—each representing 143 cm^2^ of the benthic surface—were collected with a HAPS bottom corer (KC Denmark A/S) at water depths of 12–23 m (Table S1). Each core had a diameter of 13.5 cm and was ~22–25 cm in height. From each core, 10 subsamples were taken for eDNA analyses: five subsamples from the top of the core at 0–2 cm from the sediment surface and five subsamples at 5–7 cm from the surface. Subsamples were taken with a metal teaspoon cleaned with DNA AWAY (Thermo Fisher Scientific, Carlsbad, CA, USA) and 70% EtOH before and after each new sample. Single‐use nitrile gloves and face masks were worn during sampling. As DNA from whole specimens could potentially outcompete the eDNA traces from other organisms in the sediment, we visually inspected each eDNA subsample with the naked eye, and did our best to avoid including complete specimens in the samples. These subsamples were pooled into one sediment sample of approximately 45 mL in a falcon tube, giving a total of six sediment samples from each station. Samples were immediately stored in a −20°C freezer until DNA extraction. The remainder of the core was treated as a traditional sediment core in the EPA survey (Hansen and Josefson 2014). Observations, such as sediment colour, sediment type and depth of oxygenated top sediment layer, were noted for each core (Table S1).

### 2.2 Morphological Identification of Sediment Macrofauna

Conventional sediment samples were analysed morphologically following the official Danish technical guidelines by Hansen and Josefson (2014), by a taxonomic expert who regularly performs these macrofaunal surveys for the EPA. In the laboratory, ethanol was carefully poured from the sample through a 0.5 mm mesh sieve and the sieve closely examined for any benthic macrofaunal specimens. These specimens were then visually examined and sorted using a stereo microscope. For each sample, specimens were identified to the best possible taxonomic resolution, while noting down biomass (wet weight) and number of individuals for each species.

### 2.3 DNA Extraction

All DNA extractions from sediments were carried out in a clean laboratory facility, which is dedicated to eDNA samples and other samples of low or degraded DNA content. All falcon tubes were cleaned on the outside using a 20% chlorine solution followed by 70% EtOH. Each sample was rigorously vortexed for at least 30 s to ensure homogenisation. DNA was extracted from the homogenised samples using the PowerMax Soil DNA Isolation kit (MO BIO Laboratories Inc. Carlsbad, CA, USA) following the manufacturer's protocol. We added 10 g of sample to the PowerMax Bead Solution Tube and vortexed the tube before mixing with the C1 solution. The mixture was then incubated on a vortexer for 10 min before being centrifuged, after which the supernatant was mixed with the C2 solution. An extraction control was included for each of the two extraction batches. Extracted samples and blanks were stored at −20°C until further analysis.

### 2.4 PCR Amplification, Library Building and Sequencing

PCR amplification was performed using an optimised version of the ‘Leray’ primer set (Leray et al. 2013), as described by Wangensteen et al. (2018). The optimised primer set consists of the forward primer mlCOIintF‐XT (5′‐GGWACWRGWTGRACWITITAYCCYCC‐3′) and the reverse primer jgHCO2198 (5′‐TAIACYTCIGGRTGICCRAARAAYCA‐3′), which together amplify ~313 bp of the mitochondrial COI gene. Primers were tagged with unique oligonucleotides, designed using the OligoTag program (Coissac 2012). The tags consisted of six nucleotides with at least three bases of distance between any two tags. Tags were preceded by two or three random bases; NNN or NN (de Barba et al. 2014). In order to identify errors due to tag jumps (Schnell, Bohmann, and Gilbert 2015), identical tags were used on the forward and reverse primers for each sample (Zinger et al. 2019). Four identical PCR setups were run, each containing one PCR replicate of each of the 24 samples, one PCR replicate of each extraction control and one no template control (NTC). Each of the 27 reactions in a PCR setup had a unique tag, but the same tag was used across PCR replicates of the same sample. Each PCR setup was run using 10 μL HotStarTaq Master Mix (Qiagen, Cat. no. 203445), 8 μL ddH_2_O, 1 μL BSA (Bionordica, Cat. No. B9000S), 3 μL of primer mix (5 μM forward and 5 μM reverse) and 3 μL DNA template. The thermocycler conditions for the PCR setup were set to an initial 15 min denaturation at 95°C, followed by 50 cycles of 94°C for 60 s, 45°C for 60 s and 72°C for 60 s, and a final extension of 72°C for 5 min. DNA yield and fragment sizes were checked on 2% agarose gels stained with GelRed. Equal volumes of 4 μL from each PCR reaction within a PCR setup were pooled into one tube. A library was then prepared from each of the four pools using the TruSeq DNA PCR‐free LT Sample Prep kit (Illumina) following the manufacturer's protocol. Libraries were sequenced on a NovaSeq 6000 platform by Novogene (Cambridge, UK) using 250 PE sequencing and requesting 10 Gb of output per library.

### 2.5 Bioinformatic Data Filtering

Sequencing data were processed using the MetaBarFlow pipeline (Sigsgaard et al. 2022), which uses a Python‐based workflow tool to efficiently process large metabarcoding datasets. The specific workflow and scripts used in this study are available upon request but follow default settings of MetaBarFlow. The main steps of the workflow are described in the following. Raw reads were trimmed of primers and demultiplexed using the software cutadapt (Martin 2011). Sequences were quality trimmed to an average quality score of 28 using sickle (Joshi and Fass 2011). Error modelling with the DADA2 algorithm (Callahan et al. 2016), an updated version of the DADA algorithm by Rosen et al. (2012), was applied to correct erroneous sequences. Resulting amplicon sequence variants (ASVs) were searched against a custom‐built database (https://github.com/evaegelyng/COI_database) containing COI sequences from the NCBI GenBank nt database and the Barcode of Life Data System (BOLD, https://www.boldsystems.org/). The database was built using a slightly modified version of the MARES database pipeline (Arranz et al. 2020). From the nt database, all taxids matching the search term “eukaryota[ORGN] AND species[RANK]” were identified, and all sequences matching these taxids and the search terms “(CO1[GENE] OR COI[GENE] OR COX1[GENE] OR COXI[GENE]) AND Eukaryota[ORGN] AND 2003:2022[PDAT]” were downloaded in October 2022. For BOLD, any sequences with a “markercode” of “COI‐5P” or “COI‐3P” were downloaded. Thus, we did not limit our database to marine taxa. We also did not use the custom taxonomy database or custom taxids but instead used the NCBI taxonomy with a dummy taxid for those taxa that did not have an NCBI taxid. We allowed up to 500 sequence matches per query and initially required a minimum of 90% query coverage per high‐scoring segment pair, as well as a minimum of 80% sequence similarity. Each sequence was then taxonomically classified using the R package *taxizedb* (Chamberlain and Arendsee 2020), and automatically assigned to the lowest common ancestor of all matching taxa that overlapped in their range of sequence similarities (i.e., we only assigned species‐level identifications where barcode gaps were present (Puillandre et al. 2012)). In cases where an ASV was not identified to species level, we then manually searched the sequence against the BOLD and nt databases, and updated the taxonomic classification if synonyms, typos or similar mistakes were causing the unspecific identification. For this manual curation, we used the World Register of Marine Species (WoRMS) as the authoritative taxonomy (Ahyong et al. 2024). Sequences with multiple best matches were also investigated for the distribution of the matched species, and if only one of the best matches was known to be present in Denmark, the sequence was assigned to that species. Lastly, only sequences classified as metazoans with sequence similarities of ≥ 98% and a query coverage of 100% were retained in the final dataset. When counting the total number of detected taxa, all identifications were included, but for exact‐level identifications (e.g., species, genus and family), conservative estimates were made, counting only unique taxa (e.g., *Cletodes* sp. and *Cletodes longicaudatus* count as two taxa but only one species since redundancy cannot be excluded). Bioinformatic analyses were run using the high‐performance computing facility GenomeDK, Center for Genome Analysis and Personalized Medicine, Aarhus University.

### 2.6 Diversity Analyses

ASVs occurring in a PCR blank or a PCR replicate of an extraction blank in a higher read number than in any PCR replicate of a sediment sample were excluded. Taxa only occurring in a single PCR replicate within a sample were removed from that sample. To reduce bias induced by variation in sequencing depth, PCR replicates were rarefied to the median read depth across all PCR replicates using the R package ROBITools version 0.1 (LECA 2012). PCR replicates exhibiting read counts below the median depth were kept and topped up to the median read count. PCR replicates were then aggregated per sample. Rarefaction curves for each PCR replicate were produced using the function *rarecurve* to explore whether the sequencing depth was sufficient to cover the taxonomic diversity present in each of the PCR replicates. Accumulation curves for each sample were performed using the function *specaccum* to determine whether the sampling effort was sufficient to cover the taxonomic diversity within each sample.

All detected taxa across both eDNA‐based and morphological identification were divided into four categories, indicating their lifestyle: (1) meiofauna, (2) benthic macrofauna, (3) pelagic organisms and (4) both pelagic organisms and benthic macrofauna. This allowed us to identify taxa that are not detectable with the conventional 1 mm sieve identification method (i.e., pelagic and meiofaunal taxa), and thereafter compare the two identification methods both with and without including these categories. Correctly classifying taxa detected with eDNA data as belonging to either pelagic or benthic macrofauna communities can be difficult, as the majority of them will occupy both niches during their lifespan (e.g., pelagic larval stages). In such cases, we classified the taxon as being pelagic if we did not find it realistic that the benthic stage could be captured with the conventional method.

We inferred accumulation curves of the number of detected taxa per station for (1) eDNA, (2) eDNA minus pelagic and meiofaunal taxa (henceforth referred to as ‘eDNA‐PM’) and (3) morphologically identified specimens. Six nonlinear regression models reaching asymptotes (Asymp, Gompertz, Michaelis–Menten, Logis, Lomolino and Weibull) were fitted to each of the three accumulation curves, each representing six samples within a station, using the *fitspecaccum* function. Best‐fit models were selected based on AIC values, and used to extrapolate the accumulation curves and to estimate the taxon richness asymptote. We then used the function *predict* to estimate the expected taxon detections had we taken 15 biological replicate samples instead of six. This allowed us to estimate the additional taxon detections expected from taking more samples, and to estimate the total taxon richness at each station for each method.

Using area (Ven, KBM, Samso and Hjelm) as predictor, samples were evaluated for differences in community composition by applying a permutational multivariate analysis of variance (PERMANOVA) with 999 permutations and by specifying Jaccard as the distance metric (Jaccard 1901), with the function *adonis*. Jaccard distances were chosen as we used presence/absence data. For each of the three PERMANOVA tests (eDNA, eDNA‐PM and morphologically identified specimens), we also tested for multivariate homogeneity of dispersion using the function *betadisper* and no assumptions were violated. Jaccard distances were further ordinated in two dimensions using nonmetric multidimensional scaling (NMDS) by applying the *metaMDS* function. Unreferenced functions listed in this section were all from the R package *vegan* (v.2.5–6) (Oksanen et al. 2019). A map of the sampling stations was produced using the R packages *rnaturalearth* v. 1.0.1, *rnaturalearthhires* v. 1.0.0, *sf* v. 1.0–15, *sp* v. 2.1–3, *ggplot2* v. 3.4.4, *ggspatial* v. 1.1.9 and *cowplot* v. 1.1.3. All data analyses subsequent to the taxonomic classification were performed in R v. 3.6.1 (R Core Team 2019).

## 3 Results

### 3.1 Conventional Morphological Identification of Macrofauna

A total of 112 taxa and 1397 individual specimens of macrofauna, representing 14 phyla and at least 16 classes, 43 orders, 71 families, 97 genera and 107 species, were morphologically identified in the 24 sediment samples (Table S2). Specimens of Bryozoa, Nematoda, Nemertea and Porifera were only identified at phylum level. Similarly, specimens belonging to Platyhelminthes and certain arthropod taxa were only identified to class level (i.e., Turbellaria and some Hexanauplia), as more detailed morphological identifications of these taxa are not carried out as part of the regular EPA survey. A single chordate taxon was also found, namely, a tunicate identified to the genus of *Styela*.

**FIGURE 2:**
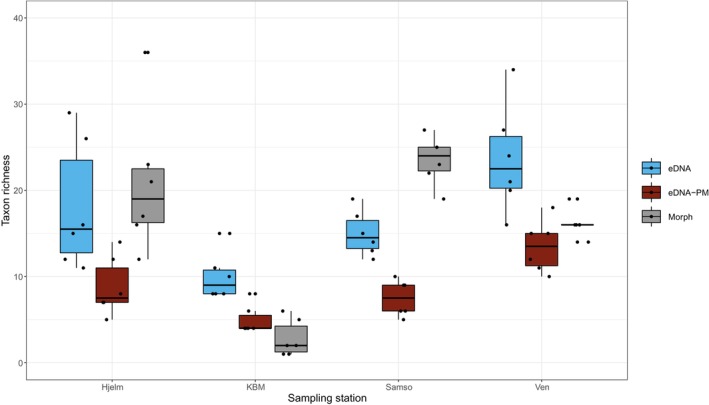
Boxplot of taxon richness for all eDNA detections (eDNA), eDNA detections excluding pelagic‐ and meiofaunal taxa (eDNA‐PM) and with conventional morphological identifications (Morph). Higher‐level taxonomic identifications are included in the taxon count in cases where no lower‐level identifications within the same taxon were found (i.e., *Cletodes* sp. would not count if *Cletodes longicaudatus* was found in the same sample).

**FIGURE 3:**
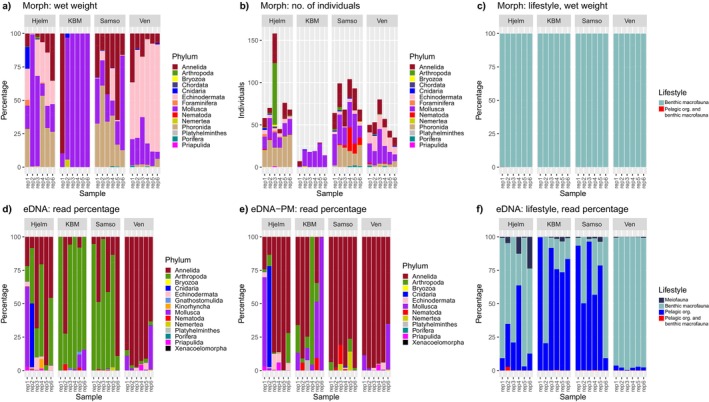
(a–c): Stacked barplots showing percentage of wet weight per phylum (a), number of individuals per phylum (b) and percentage of wet weight per lifestyle category (c), using conventional morphological identification. (d–f): Stacked barplots showing relative eDNA read counts per phylum (d), per phylum when excluding pelagic‐ and meiofaunal taxa (e) and per lifestyle category (f).

Of the four stations, Samso had the highest taxon richness, whereas KBM had the lowest (Figure 2). The taxonomic composition was, at the phylum level, dominated by annelids (50%), but molluscans (22%), echinoderms (8%) and arthropods (8%) were also represented (Table S2). In terms of wet weight, molluscs, annelids, echinoderms and phoronids generally dominated the samples (Figure 3a). Molluscs were particularly dominant at KBM, while echinoderms contributed the most at Ven, and Hjelm and Samso were more influenced by phoronids. In terms of numbers of individuals, KBM clearly stood out with the fewest individuals per replicate (Figure 3b). Nearly all identified taxa across stations were Category 2 (benthic macrofauna, Figure 3c), with only *Hydrozoa* sp. and *Copepoda* sp. treated as Category 4 (both pelagic organisms and benthic macrofauna).

### 3.2 Taxonomic Diversity Obtained From eDNA

A total of 227.8 M raw reads corresponding to 113.9 M read pairs were generated. We obtained a similar sequencing depth across the four libraries with 24.4–32.4 M read pairs per library (average of 28.5 M read pairs per library). After initial merging, filtering and trimming, a total of 0.4 –3.0 M reads per sample were retained (average of 1.2 M reads per sample, *n* = 24). The two extraction controls retained 55,104 and 8 reads, respectively, whereas the NTC retained 501 reads in total. Overall, we obtained 78,153 ASVs, of which 810 (1.04%) were taxonomically identified as metazoans and had a minimum of 98% similarity to and 100% query coverage of the best database match. After all filtration steps, median read count per PCR replicate was 61,498 reads, and after aggregating rarefied replicates, each sample contained a total of 245,988 reads. These reads represented 107 taxa, distributed across 14 phyla. Seven of the 107 taxa fell under the criterion of redundancy (see methods), leading to a minimum estimate of 100 unique species, representing at least 26 classes, 46 orders, 62 families and 74 genera (Table S2).

*Homo sapiens* was detected both in NTCs and in extraction controls, but not in any experimental samples. The remaining reads in the NTCs were of unspecific origin. Four reads (a single ASV) of *Prionospio fallax* were found in an extraction control, but the species was kept in the dataset as its highest read count in a sample was 326,785 reads.

Rarefaction curves for individual PCR replicates revealed a saturation of metazoan richness, indicating sufficient sequencing depth (Figure S1). The species accumulation curves showed that four PCR replicates were more than sufficient to cover the common diversity within each sample (Figure S1).

Based on eDNA, Ven had the highest taxon richness of the four stations, while KBM had the lowest (Figure 2). At the phylum level, arthropods were dominant in terms of their proportion of total taxa (33%), but annelids (27%), molluscans (12%), cnidarians (10%) and echinoderms (7%) were also well represented (Figure 3d and Table S2).

We identified 13 taxa with pelagic lifestyles in the eDNA data; 10 arthropods, two cnidarians and one nematode (Table S2). Another 22 taxa were identified as meiofauna; 19 arthropods, the majority of which belonged to the order Harpacticoida, one kinorhynch, one gnathostomulid and one nematode (Table S2). Removing taxa classified as either pelagic or meiofaunal resulted in annelids being the dominant phylum (40%), followed by molluscans (18%), cnidarians (13%) and echinoderms (10%) (Figure 3e). Most of the sequencing reads classified according to lifestyle were found to represent benthic macrofauna at the stations Hjelm and Ven, whereas pelagic organisms contributed the most reads at KBM and Samso (Figure 3f).

### 3.3 Complementarity of eDNA Metabarcoding and Conventional Morphological Identification

Observed taxon richness varied among sampling stations, samples and identification methods (Figure 2), but both methods found KBM to have the lowest richness. No method was clearly superior to the other in detecting the most unique taxa per station, with eDNA identifying the most taxa of the two methods at Ven (eDNA = 57, eDNA‐PM = 35, Morph = 36) and KBM (eDNA = 24, eDNA‐PM = 15, Morph = 9), and the conventional method identifying the most taxa at Hjelm (eDNA = 45, eDNA‐PM = 26, Morph = 57) and Samso (eDNA = 39, eDNA‐PM = 22, Morph = 59).

**FIGURE 4:**
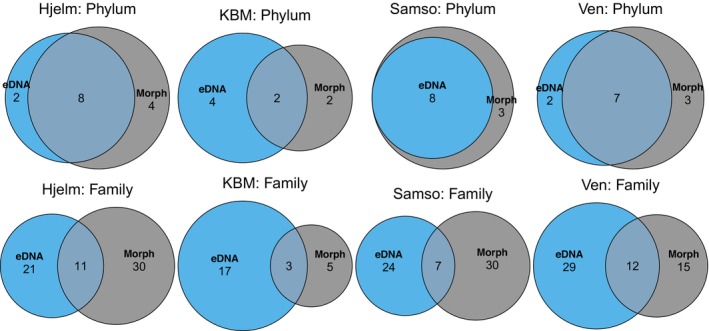
Venn diagrams of phylum‐level and family‐level overlap between eDNA and morphological identification for each of the four sampling stations. Only taxa identified to the respective levels are included.

We found only a slight taxonomic overlap between the two methods, with 26 taxa shared out of 107 and 112 taxa for the eDNA and the conventional method, respectively (Figures S3 and S4). Approximately 32% of the species that were identified with the conventional method, but not with eDNA, were simply not possible to identify to species level with eDNA, due to missing or partial reference sequences of COI in the BOLD and GenBank databases (Table S2). At the phylum level, we found an overlap of 11 phyla, but the phyla Kinorhyncha, Gnathostomulida and Xenacoelomorpha were found only with eDNA, and the phyla Foraminifera, Phoronida and Chordata were found only with the conventional method (Table S2). At the family level, we found an overall overlap of 26 families, but 38 families were only found with the conventional method and 45 (24 with the eDNA‐PM dataset) families were only found with eDNA (Figure S5). Station‐specific family‐level comparisons also revealed little overlap (3–12 families) between eDNA and morphological sampling, with much higher numbers of families found exclusively with one method (17–29 for eDNA; 5–30 for morphological sampling) (Figure 4).

**FIGURE 5:**
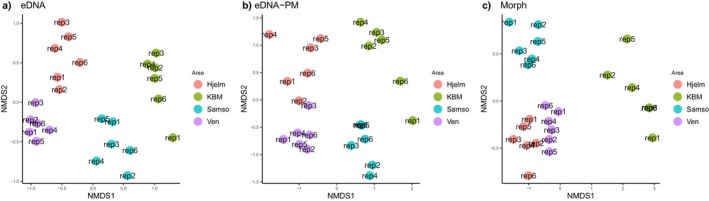
Nonmetric multidimensional scaling (NMDS) plot of biological communities using Jaccard distances for eDNA (a), eDNA excluding pelagic‐ and meiofaunal taxa (eDNA‐PM) (b) and conventional morphological data (c).

Both eDNA and conventional approaches showed taxon compositions that distinguished the four sampling stations (Figure 5). For eDNA, this was true both with and without meiofauna and pelagic taxa, but the pattern was slightly more distinct in the former case, which also resulted in a clearer clustering of samples from the same station (Figure 5a,b). The PERMANOVA test showed that the area sampled could explain 45%, 41% and 49% (*p* < 0.01) of the variation in community composition for eDNA, eDNA‐PM and morphological data, respectively. Ordination analyses for both methods placed KBM furthest to the right on the first axis, whereas no consistent pattern was found between the two methods for the other three sampling stations.

### 3.4 Extrapolated Taxon Richness

**FIGURE 6:**
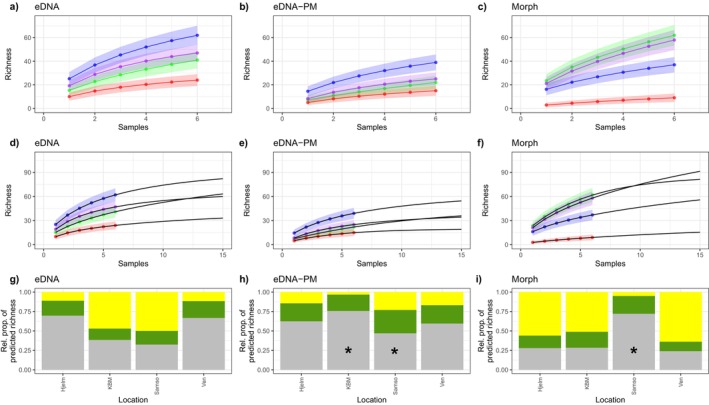
(a‐c): Accumulation curves of detected taxa per sampling station for eDNA, eDNA detections excluding pelagic‐ and meiofaunal taxa (eDNA‐PM) and morphological identification (Morph). (d‐f): Extrapolation of accumulation curves using nonlinear regression best‐fit models (Weibull and Asymp) to an arbitrary 15 samples. (a‐f): Colours reflect sampling stations, with Hjelm (purple), KBM (red), Samso (light green) and Ven (blue). (g‐i): Overview of the relative proportion of the predicted richness (asymptote of best‐fit model) covered by taking six samples (grey), the additional expected gain by taking 15 samples (green), and the remaining undetected proportion of the predicted richness (yellow). Note that three of the 12 data series had Asymp as the best model fit (KBM and Samso for eDNA‐PM and Samso for morphological identification, marked by an asterisk), whereas Weibull was the best model fit for the remaining nine data series.

Overall, the accumulation curves of detected taxa indicated that with a higher sampling effort, we could expect to detect more taxa, especially for the morphological identification approach (Figure 6a–i). Weibull was found to be the best‐fit model for most combinations of station and dataset, but in three of 12 instances, the Asymp model performed the best. The Asymp model generally reached the asymptote faster than the Weibull model, indicating that the three fits to this model likely overestimate the proportion of total richness normally captured with 6 and 15 samples, respectively. When looking at the full eDNA dataset, the highest proportion of estimated total richness was detected at Hjelm and Ven (70 and 67%, respectively, Weibull model). For the eDNA‐PM dataset, the highest proportion of estimated total richness was found at KBM (76%), although this was inferred with the Asymp model. For samples based on morphological identification, we clearly detected the highest proportion of estimated total richness at the sampling station Samso (72%), although again this was based on the Asymp model. The sampling stations Hjelm, KBM and Ven only yielded 28%, 28% and 24% of the estimated total richness, respectively, when applying the Weibull model.

## 4 Discussion

Environmental DNA analyses of marine sediments are increasingly being developed and implemented in the assessment of benthic communities. Here, we show that even when analysing the exact same sediment core using eDNA and conventional morphological identification, highly contrasting taxon compositions are found. By categorising detected taxa according to their lifestyle, we found that a nonnegligible proportion of the difference in taxon composition was explained by the detection of pelagic and meiofaunal species with eDNA, organisms which are outside the scope of conventional sampling.

### 4.1 Environmental DNA Metabarcoding

We detected a wide range of marine benthic macroinvertebrate species with eDNA that are often found with conventional methods, but pelagic and meiofaunal species also contributed substantially to the taxon richness (~33%) and read counts (~43%). The high proportion of pelagic taxa (~12%) found in the sediment by eDNA is in contrast to findings from the deep sea (> 1000 m depth), where Laroche et al. (2020) found that < 2% of the ASVs in sediment samples were pelagic species. This could indicate that benthic communities in coastal areas receive larger eDNA input from the water column than in the deep sea, perhaps due to shorter sedimentation distances and presumably less time for degradation of DNA. Importantly, the pelagic taxa that we found generally correspond well with existing knowledge. For instance, the high proportion of pelagic calanoid copepod taxa found in the current study likely reflects the influence of the spring bloom, where calanoids are known to thrive (Monrad 2021).

Interestingly, the community composition at Ven did not change dramatically when removing pelagic and meiofaunal species from the eDNA data (Figure 3d,e), as arthropods contributed very little to the read percentages at this site. Ven is located in the Sound between Denmark and Sweden, where trawling has been banned since 1932 given its importance as a busy shipping route (ICES 2022). We speculate that a potential higher integrity of the benthic fauna due to the long‐term trawl ban could have dampened the relative eDNA contributions from pelagic organisms at this site. However, there could also be other explanations related to ocean bathymetry, seasonality of pelagic blooms or even stochasticity, given the small sample size investigated here.

We found that a high proportion (32%) of the species found by conventional methods and not by eDNA could not be detected molecularly due to missing reference sequences. Lanzén et al. (2021) found a similar proportion (~22%) of benthic species that were missing reference sequences in GenBank. However, we can only speculate whether these species would actually have been found, had their reference barcodes been available. This represents an important shortcoming of eDNA metabarcoding and a potential explanation for the incongruence between our molecular and morphology‐based results. We, therefore, urge the continuous sequencing of reference material of marine metazoans as a priority in the coming years, either through national sequencing initiatives (e.g., Margaryan et al. 2020) or through consolidated large‐scale genome‐sequencing projects (Lewin et al. 2018, 2022). The choice of primers likely also affected the detection of benthic macrofauna, as even broad generic primers are known to exhibit primer bias for certain taxa (Deagle et al. 2014). Using several primer sets would likely increase the diversity obtained from eDNA, not just because of primer bias but because the chance of a reference sequence being available would increase.

The relatively small overlap between species found with eDNA and morphological methods could also be partly due to an insufficient amount of input sediment for the DNA extraction and thus an insufficient representation of the eDNA present in the sediment sample (Gielings et al. 2021). However, rarefaction and accumulation curves did not indicate this (Figures S1 and S2), and while most extraction kits only allow for low amounts of substrate input (0.2–2 g), the PowerMax Soil DNA Isolation kit used in this study allowed us to use 10 g of sediment. It is the only commercially available kit to process such a high amount of input (Pawlowski et al. 2021). Whether extracting the entire sediment sample instead of 10 g provides a more complete overview per sample should be easy to test, but would naturally increase the price and workload associated with the laboratory work. The same would be the case for the approach suggested by Pawlowski et al. (2021) to perform multiple DNA extractions per sediment sample.

### 4.2 Remarkable Findings

We detected two species with eDNA whose presence must be considered questionable. *Pisidium amnicum* , which was detected in KBM, is a small mussel that is rare in Scandinavia and has been reported exclusively from running freshwater, that is, streams and rivers. The finding likely represents an error, as it is based on a single sequence hit, and should be interpreted with caution. Similarly, we detected the nemertean species *Carinina ochracea* in a single sample at Samso. Information on the distribution of this species is extremely sparse. In Scandinavia, it is only known from Tjärnö (Sweden), which is also the type locality for the species. In addition, it is known from a few French localities (Fernández‐Álvarez, García‐Jiménez, and Machordom 2015). Whether the finding in the present study is an indication that the species has a less abrupt distribution than previously believed, must await future investigations. Unexpected species have previously been detected by eDNA and later been found to be physically present in Denmark, for example, the European pilchard ( *Sardina pilchardus* ) (Thomsen et al. 2012).

We also detected the invasive spionid polychaete *Marenzelleria viridis* using eDNA. This species has become established in Scandinavia and Denmark, after having gone largely unnoticed over the past decades (Kube et al. 1996). A study conducted in the Baltic Sea revealed that the species actually comprises a species complex consisting of three distinct species (*M. arctia*, *M. viridis* and *M. neglecta* ) (Blank et al. 2008). The existence of this species complex has introduced confusion in both morphological and molecular identification, resulting in numerous publications and molecular databases erroneously referring to all three species as *M. viridis* . It is therefore difficult to confidently determine which of the three *Marenzelleria* species we have detected. Nevertheless, it is evident that we successfully detected the presence of an invasive species using eDNA, which was not identified with the morphological investigations. This finding highlights the value of eDNA metabarcoding as a tool for observing rare, new and/or invasive species in the marine environment. This is particularly interesting as the method can provide early warnings of species that in the future may impact the local marine environment and species composition (Olsen et al. 2008).

We detected two parasitic species, namely, *Rhodinicola elongata*, an ectoparasitic copepod found on Maldanid polychaetes (bamboo worms), and *Hysterothylacium aduncum*, an endoparasitic nematode. The identification of *R. elongata* underscores the utility of traditional taxonomic methods in cases where barcodes are missing from the reference database, as species missing barcodes are not possible to identify using eDNA. Conversely, *H. aduncum* was exclusively identified with eDNA, demonstrating the promising application of eDNA techniques for detecting endoparasites that might elude conventional methods. Parasites are frequently underrepresented in taxonomic biodiversity investigations, despite their substantial contribution to overall biodiversity and their profound ecological impact (Dobson et al. 2008). The inherent oversight of parasites is noteworthy given their potential to significantly influence habitat dynamics (Kuris et al. 2008). This finding exemplifies the evolving significance of eDNA as an economical and robust tool for uncovering overlooked facets of marine biodiversity. Specifically within the realm of parasitology, qualitative and semiquantitative eDNA analyses of parasite diversity could yield valuable insights into potential stress levels in marine habitats, a topic that has not yet been thoroughly explored.

### 4.3 Pros and Cons of the Two Identification Methods

The obvious advantage of using the classical morphological identification method for environmental monitoring is the possibility of quantifying numbers of individuals or biomass. Currently, such information is not yet obtainable using eDNA methods (Danziger, Olson, and Frederich 2022), although some genetic markers indicate promising correlations between metabarcoding read counts and invertebrate biomass estimates under controlled experimental conditions (Schenk et al. 2019). Recent work using mock samples to calibrate for PCR bias has also shown considerable promise for deriving quantitative estimates (Guri et al. 2024; Shelton et al. 2023). Nonetheless, the visual confirmation of an organism's presence provides a higher level of certainty than the presence of its DNA and can also provide data on its physical state (e.g., dead or alive, healthy or diseased, adult or juvenile, male or female). In contrast, traces of eDNA are not necessarily linked to living organisms and—at least with current methods—do not provide any information regarding the physical state of the organism at hand (Ellegaard et al. 2020; Sigsgaard et al. 2020).

However, eDNA does arguably provide a more objective way to estimate the biodiversity of a given sediment sample, while morphological methods are likely more biased by interpersonal variation in identification skills and sorting experience (Troudet et al. 2017). Additionally, certain taxa can be very difficult and/or time consuming to identify morphologically, often resulting in higher‐level taxonomic identifications. A prominent example of this is Nematoda, which constitutes a species‐rich group in marine sediments, but whose representatives are rarely identified further than the phylum level in morphological samples (Pantó et al. 2021). In our study, genus‐ or species‐level identification could be made for three nematode taxa based on eDNA, whereas only ‘*Nematoda* sp.’ was registered using morphology. Furthermore, the grabbing, sifting and sorting procedures that precede the morphological identification can be damaging to soft‐bodied animals, and thereby often prohibitive of taxonomic identification (Aylagas et al. 2018). As a potential example of this, a sea sponge was identified to species level ( *Cliona celata* ) with eDNA, but only ‘*Porifera* sp.’ was registered based on morphology, which might be due to only fragments of the organisms being left in the sample. Alternatively, *C. celata* might have gone unnoticed as boring sponges can hide inside molluscan shells. Similarly, larval stages can be virtually impossible to separate out physically in a nondestructive way and to identify to species level. In contrast, as eDNA found in sediments is independent of the physical state and life stage of the collected organisms, it arguably offers more detailed insights into biodiversity by allowing identification of organisms to lower taxonomic levels, albeit without distinguishing between dead and live organisms and without direct quantification.

### 4.4 Complementarity of eDNA Metabarcoding and Conventional Morphological Sampling

In general, we found a low congruence between results from eDNA metabarcoding and conventional morphological identification obtained from the same sediment samples (Table S2, Figure 4). Disparities were to some degree explained by the lifestyle of the detected species. When pelagic and meiofaunal species were removed from the comparison, the two datasets became slightly more similar at phylum, family and species level (Table S2, Figure 3a,e).

We also found inconsistencies between the morphological and eDNA data that could perhaps be explained by taxonomic misidentification. For example, the invasive polychaete *M. viridis* (see above) was identified by eDNA in a sediment sample from KBM. In the corresponding morphological data, however, the species *Polydora cornuta* of the same family (Spionidae) was found instead. As multiple DNA reference sequences were available for both species, the eDNA‐based detection of *M. viridis* is most likely correct, and the observation of *P. cornuta* could reflect a morphological misidentification of *M. viridis* . However, we were not able to pursue a reidentification of the specimen in question.

An important caveat of our comparative study is that the NOVANA biomonitoring programme only identifies macrofauna retained on a 1 mm sieve. This mesh size may miss juvenile macrofauna, and as such we might be targeting somewhat different communities with our two approaches.

Overall, our results provide additional insight into the respective strengths and weaknesses of analysing sediment samples with eDNA versus with morphology and support the complementarity of the methods. We therefore support previous recommendations that these approaches should preferably be used in parallel for inferring taxon composition and richness in benthic monitoring surveys. However, while the taxon compositions detected with these two different approaches might not indicate much overlap, they may still result in similar biomonitoring conclusions (Aylagas et al. 2018). Depending on the metrics (i.e., species composition, abundance or biomass) taken into consideration for existing marine benthic biomonitoring programmes, we advocate that future efforts should be directed towards investigating the extent to which monitoring conclusions based on eDNA sampling and classic morphology‐based methods agree.

### 4.5 Future Perspectives

In face of environmental change, we need cost‐efficient methods for mapping and monitoring patterns of marine biodiversity to better understand how it responds, and to make more comprehensive and reliable impact assessments. Environmental DNA metabarcoding approaches are increasingly relevant to consider in this context, with public genetic databases rapidly expanding and with plummeting sequencing costs. As shown in this and previous studies, eDNA often provides a complementary taxon list to conventional methods and can thus broaden and strengthen the analyses and conclusions made from monitoring. In benthic sediments, this might entail detection of cryptic species or juvenile stages, but also, as our results show, pelagic and meiofaunal species. Interestingly, we found that eDNA requires less biological replication for maximum coverage of diversity to be reached—although importantly, both methods appear to require a higher level of replication than generally applied to reach their full potential. Lastly, taxonomy‐free analyses of eDNA data have recently shown great promise for marine ecosystem assessment (Cordier et al. 2018; Frühe et al. 2021; Frühe et al. 2021). While taxonomic identification to a low level is still preferable, due to the ability to draw on existing ecological knowledge, this novel approach largely overcomes the limitations of current reference databases and constitutes a major step towards unlocking the full potential of eDNA metabarcoding. In conclusion, we recommend that eDNA analyses of sediment and morphological identification of benthic macrofauna are used in concert for marine bioassessment, and that as many biological and technical replicates as feasible are collected until an appropriate replication level for the specific context can be determined.

## Author Contributions

**Mads Reinholdt Jensen:** data curation (lead), formal analysis (lead), software (lead), visualization (lead), writing – original draft (equal), writing – review and editing (lead). **Sune Agersnap:** conceptualization (equal), formal analysis (supporting), investigation (lead), methodology (equal), writing – original draft (equal), writing – review and editing (supporting). **Eva Egelyng Sigsgaard:** methodology (equal), software (supporting), supervision (supporting), validation (equal), writing – original draft (supporting), writing – review and editing (supporting). **Marcelo de Paula Ávila:** methodology (supporting), software (supporting), visualization (supporting), writing – original draft (supporting). **Henrik Glenner:** formal analysis (supporting), methodology (supporting), supervision (supporting), writing – original draft (supporting), writing – review and editing (supporting). **Mary S. Wisz:** supervision (supporting), writing – original draft (supporting), writing – review and editing (supporting). **Philip Francis Thomsen:** conceptualization (equal), funding acquisition (lead), methodology (equal), project administration (lead), resources (lead), supervision (lead), writing – original draft (supporting), writing – review and editing (supporting).

## Conflicts of Interest

The authors declare no conflicts of interest.

## Supporting information


Data S1.

## Data Availability

Raw sequencing data and morphological identification data, as well as output files from running MetaBarFlow, are available on Dryad. The data can be accessed via https://doi.org/10.5061/dryad.mw6m9065b https://datadryad.org/stash/share/R16PE7fRJ9H4Hoaxl1nL0m‐riX8wtC8Oiup‐ll56tjI. Any enquiries should be directed to the corresponding authors.
