# Supplementary material for: The Core of the Matter—Importance of Identification Method and Biological Replication for Benthic Marine Monitoring
Source: Ecol Evol. 2024 Nov 14;14(11):e70556. doi: 10.1002/ece3.70556 (PMC11563695; doi:10.1002/ece3.70556)
Supplement: Supplementary file 1 — Data S1. [file ECE3-14-e70556-s001.docx]

SUPPORTING INFORMATION

for

**The core of the matter – Importance of identification method and biological replication for benthic marine monitoring**

Mads Reinholdt Jensen^1,2†*^, Sune Agersnap^1†^, Eva Egelyng Sigsgaard^1,3^, Marcelo De Paula Avila^1^, Henrik Glenner^4,5^, Mary Suzanne Wisz^6^, Philip Francis Thomsen^1*^

1. *Department of Biology, Aarhus University, Ny Munkegade 116, Building 1540, DK-8000 Aarhus C, Denmark*
2. *Norwegian College of Fishery Science, UiT – The Arctic University of Norway, Tromsø, Norway*
3. *Center for Sustainable Landscapes under Global Change (SustainScapes), Aarhus University, Aarhus C, Denmark.*
4. *Department of Biological Sciences, University of Bergen, Bergen, Norway*
5. *Center of Macroecology and Climate, GLOBE, University of Copenhagen, Copenhagen, Denmark*
6. *Ocean Sustainability, Governance and Management, World Maritime University, Malmö, Sweden*

† These authors contributed equally to the work

* Corresponding authors. ORCID: 0000-0001-8240-1083. Tel.: +45 21 68 36 18, E-mail address: mads.jensen@uit.no (M. R. Jensen). ORCID: 0000-0002-9867-4366. Tel.: +45 27 14 20 46, E-mail address: pfthomsen@bio.au.dk (P. F. Thomsen).

**Figure S1:** Rarefaction curves per PCR replicate per sample, indicating that sequencing depth was sufficient for the PCR replicates sequenced. The individual plots show the

accumulated number of species (y-axis) recovered as a function of sequencing depth (x-axis)

for each sample. The four PCR replicates are numbered with a running number.


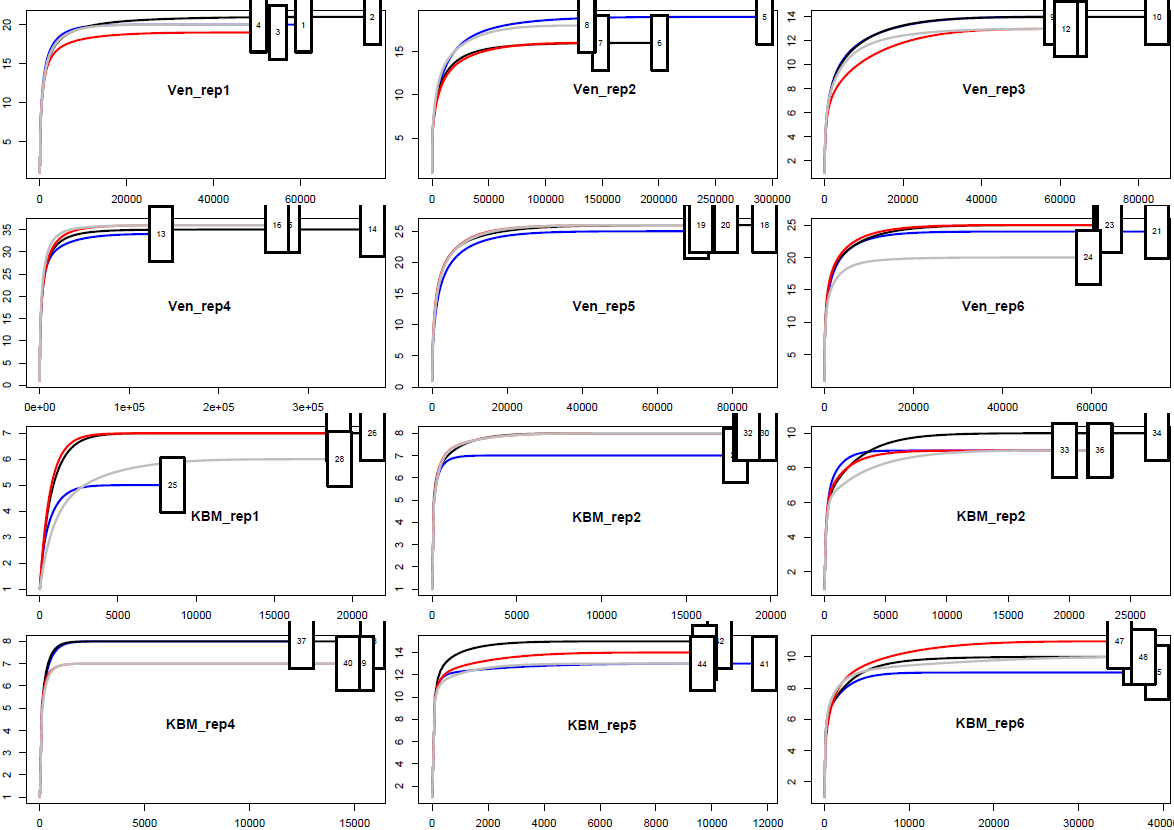


**Figure S1 (continued):** Rarefaction curves per PCR replicate per sample, indicating that sequencing depth was sufficient for the PCR replicates sequenced. The individual plots show the accumulated number of species (y-axis) recovered as a function of sequencing depth (x-axis) for each sample. The four PCR replicates are numbered with a running number.


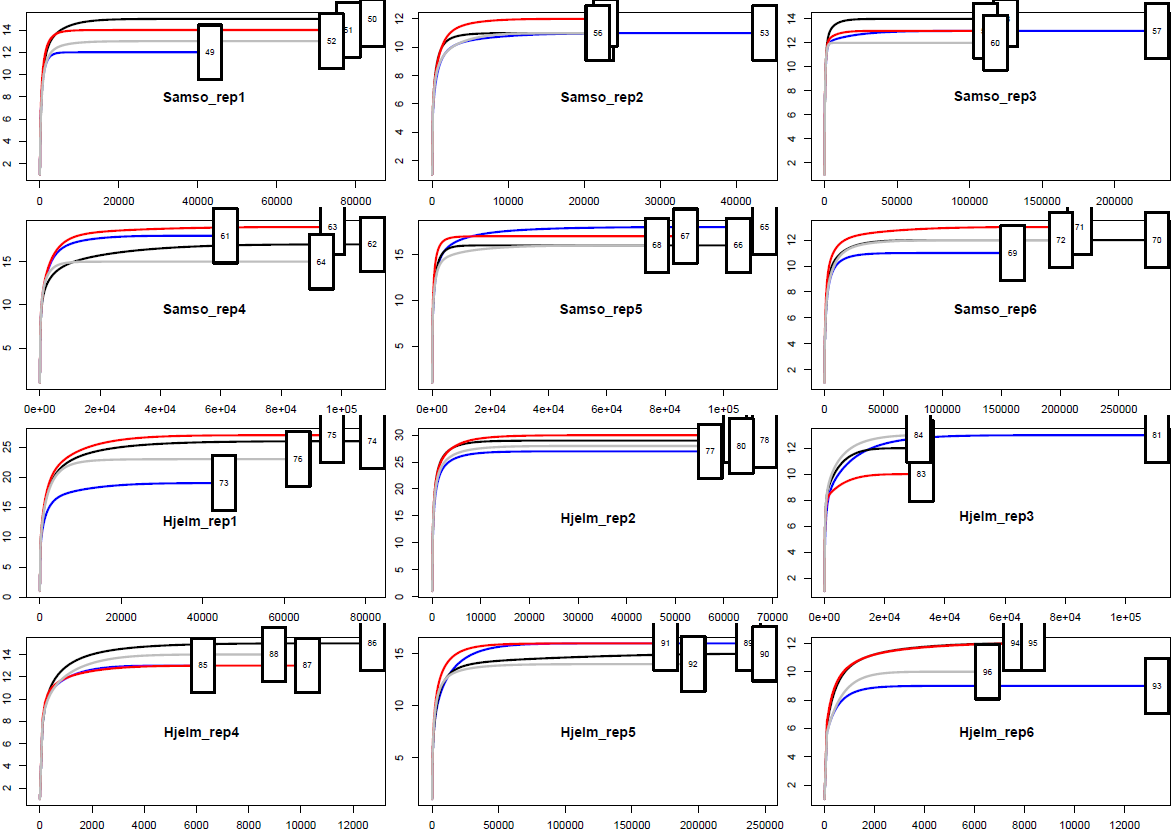


**Figure S2:** Species accumulation curves, indicating that four PCR replicates were enough to cover the diversity in each sample. Y-axis represents number of species found and X-axis represents PCR replicates with confidence intervals shown as light blue.


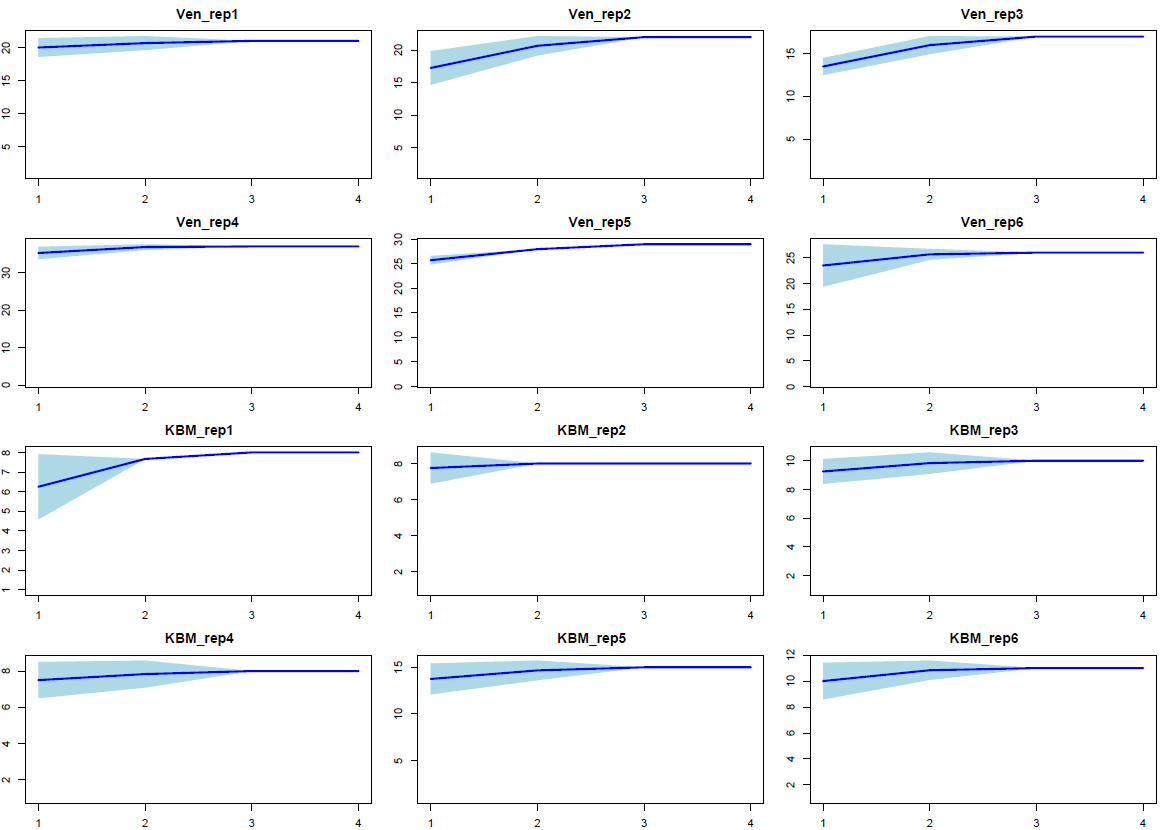


**Figure S2 (continued):** Species accumulation curves, indicating that four PCR replicates were enough to cover the diversity in each sample. Y-axis represents number of species found and X-axis represents PCR replicates with confidence intervals shown as light blue.


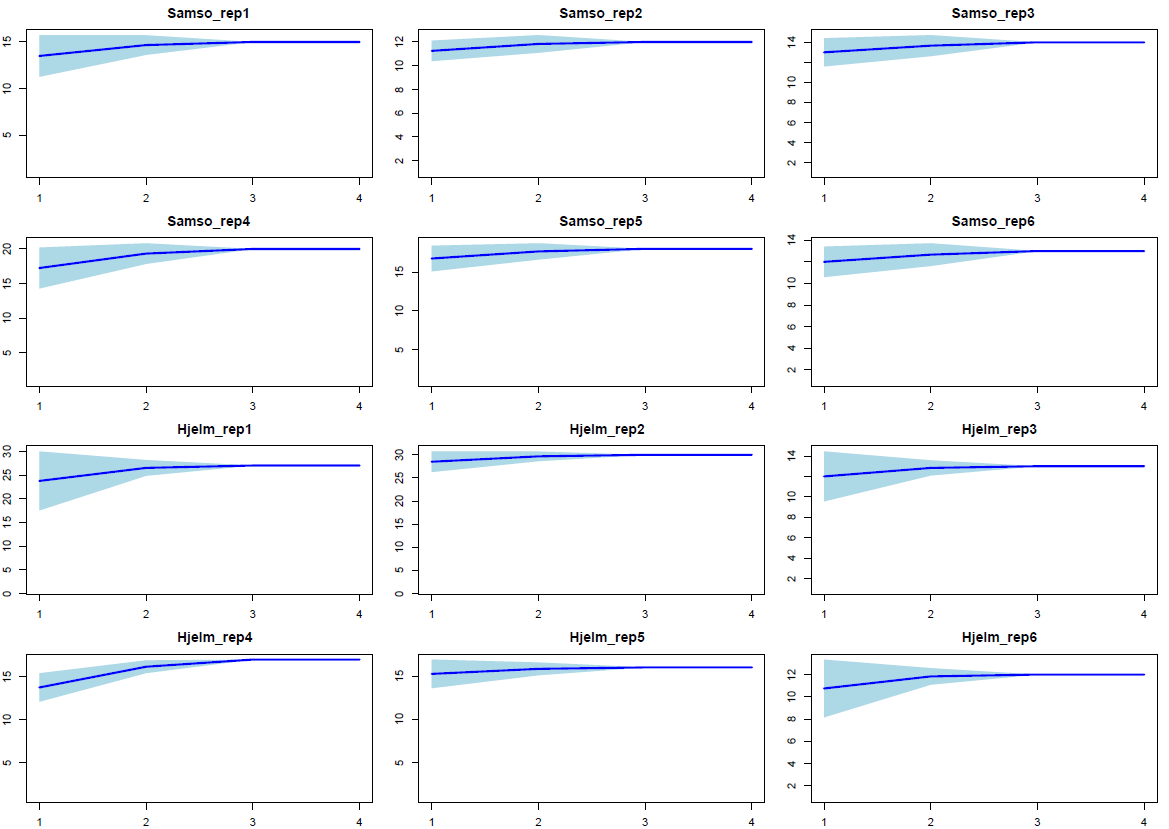


**Figure S3:** Heatmap of all taxa detected in this study (presence/absence) after filtering and rarefaction. Data from the six samples taken from each location has been merged, and any taxon present in either of the samples is included here. Rows are colour-coded according to the type of sample (“eDNA” or “Morph”) and location (“Ven”, “KBM”, “Samso”, and “Hjelm”). Columns are colour coded according to the lifestyle groups used (1=meiofauna, 2=benthic macrofauna, 3=pelagic organisms and 4=both pelagic organisms and benthic macrofauna). Note that many of these organisms have pelagic larvae but infaunal adult stages, and that eDNA is not able to distinguish between the two. Here, we have assigned species detected by eDNA as “benthic macrofauna” if we found it realistic that they would be detected by the morphological approach.
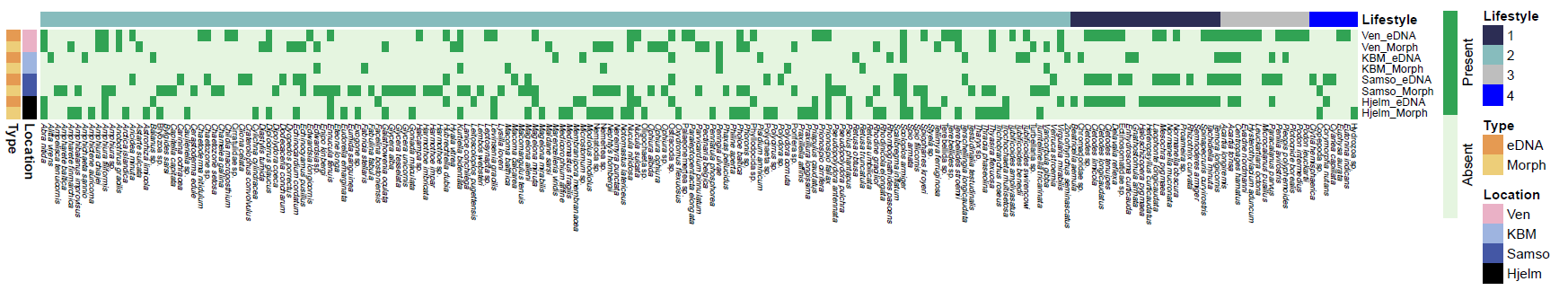


**Figure S4:** Heatmap of all taxa detected in this study (presence/absence) after filtering and rarefaction for each individual sample. Rows are colour-coded according to the type of sample (“eDNA” or “Morph”) and location (“Ven”, “KBM”, “Samso”, and “Hjelm”). Columns are colour coded according to the lifestyle groups used (1=meiofauna, 2=benthic macrofauna, 3=pelagic organisms and 4=both pelagic organisms and benthic macrofauna). Note that many of these organisms have pelagic larvae but infaunal adult stages, and that eDNA is not able to distinguish between the two. Here, we have assigned species detected by eDNA as “Infauna” if we found it realistic that they would be detected by the morphological approach.
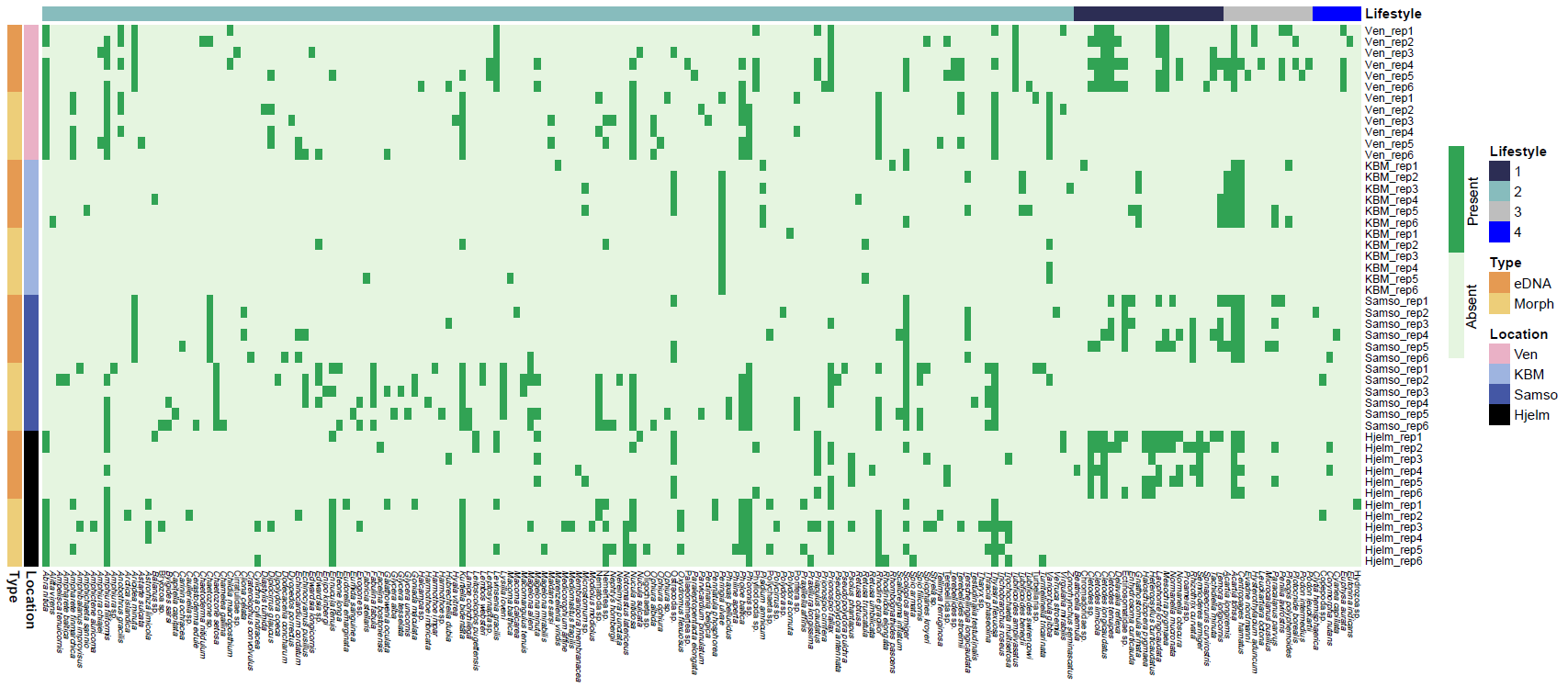


**Figure S5:** Venn diagrams of phylum-level and family-level overlap between eDNA and morphological identification across all sites combined. Only taxa identified to the respective levels are included.


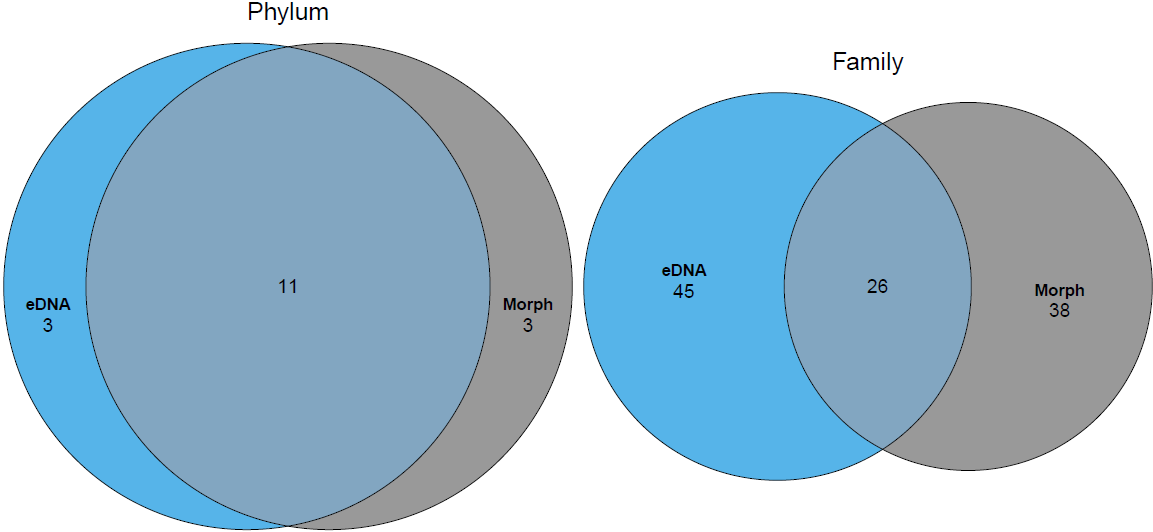


| **Table S1:** A sampling overview for the 24 collected sediment cores at the four sampling stations, including metadata and observations of color, depth of oxygenated top layer, smell and type of sediment (D = dominant, O = occasional). | | | | | | | | | | | |  |
| --- | --- | --- | --- | --- | --- | --- | --- | --- | --- | --- | --- | --- |
| **Sample name** | **Location** | **Station name** | **Sample date (d/m/y)** | **Lat.** | **Lon.** | **Depth (m)** | **Core height (cm)** | **Sediment color** | **Oxyg. top layer (mm)** | **Sediment smell** | **Sediment type** | |
| KBM_rep1 | Karrebaeksminde bugt | BF | 21/3/19 | 55.0877 | 11.331 | 12.7 | 23 | Brownish | 2 | Heavy smell | Silt (D), sand and clay (O) | |
| KBM_rep2 | Karrebaeksminde bugt | BF | 21/3/19 | 55.0083 | 11.2783 | 13 | 23 | Brownish | 2 | Heavy smell | Silt (D), sand and clay (O) | |
| KBM_rep3 | Karrebaeksminde bugt | BF | 21/3/19 | 55.0834 | 11.3155 | 12.8 | 22 | Brownish | 1 | Heavy smell | Silt (D), sand and clay (O) | |
| KBM_rep4 | Karrebaeksminde bugt | BF | 21/3/19 | 55.0787 | 11.3096 | 12.9 | 23 | Brownish | 1 | Heavy smell | Silt (D), sand and clay (O) | |
| KBM_rep5 | Karrebaeksminde bugt | BF | 21/3/19 | 55.0748 | 11.2856 | 13.4 | 22 | Brownish | 2 | Heavy smell | Silt (D), sand and clay (O) | |
| KBM_rep6 | Karrebaeksminde bugt | BF | 21/3/19 | 55.0747 | 11.33 | 12.8 | 22 | Brownish | 2 | Heavy smell | Silt (D), sand and clay (O) | |
| Hjelm_rep1 | Hjelm | HSD P23 | 24/4/19 | 56.0757 | 10.57924 | 23.2 | 25 | Greyish | > 0,5 | None | Silty clay/clay (D) | |
| Hjelm_rep2 | Hjelm | HSD P23 | 24/4/19 | 56.0766 | 10.57754 | 23.2 | 25 | Greyish | > 0,5 | None | Silty clay/clay (D) | |
| Hjelm_rep3 | Hjelm | HSD P23 | 24/4/19 | 56.0765 | 10.57763 | 23.2 | 25 | Greyish | > 0,5 | None | Silty clay/clay (D) | |
| Hjelm_rep4 | Hjelm | HSD P23 | 24/4/19 | 56.0765 | 10.57777 | 23.2 | 25 | Greyish | > 0,5 | None | Silty clay/clay (D) | |
| Hjelm_rep5 | Hjelm | HSD P23 | 24/4/19 | 56.0767 | 10.57802 | 23.2 | 25 | Greyish | > 0,5 | None | Silty clay/clay (D) | |
| Hjelm_rep6 | Hjelm | HSD P23 | 24/4/19 | 56.0768 | 10.57794 | 23.2 | 25 | Greyish | > 0,5 | None | Silty clay/clay (D) | |
| Samso_rep1 | Samso | HSD 14 | 24/4/19 | 55.5883 | 10.48601 | 17.5 | 25 | Brownish | < 5 | None | Sand (D), silt/clay (O) | |
| Samso_rep2 | Samso | HSD 14 | 24/4/19 | 55.5884 | 10.48594 | 17.5 | 25 | Brownish | < 5 | None | Sand (D), silt/clay (O) | |
| Samso_rep3 | Samso | HSD 14 | 24/4/19 | 55.5884 | 10.48637 | 17.5 | 25 | Brownish | < 5 | None | Sand (D), silt/clay (O) | |
| Samso_rep4 | Samso | HSD 14 | 24/4/19 | 55.5885 | 10.48624 | 17.5 | 25 | Brownish | < 5 | None | Sand (D), silt/clay (O) | |
| Samso_rep5 | Samso | HSD 14 | 24/4/19 | 55.5885 | 10.48590 | 17.6 | 25 | Brownish | < 5 | None | Sand (D), silt/clay (O) | |
| Samso_rep6 | Samso | HSD 14 | 24/4/19 | 55.5885 | 10.48585 | 17.6 | 25 | Brownish | < 5 | None | Sand (D), silt/clay (O) | |
| Ven_rep1 | Ven | HSD 31S | 1/4/19 | 55.5107 | 12.4002 | 19.9 | 22 | Light brown | 1 | None | Silt (D), clay (O) | |
| Ven_rep2 | Ven | HSD 31S | 1/4/19 | 55.5124 | 12.3981 | 18.7 | 22 | Light brown | 1 | None | Silt (D), clay (O) | |
| Ven_rep3 | Ven | HSD 31S | 1/4/19 | 55.5136 | 12.3975 | 17.9 | 22 | Light brown | 3 | None | Silt (D), clay (O) | |
| Ven_rep4 | Ven | HSD 31S | 1/4/19 | 55.5103 | 12.4017 | 19.5 | 23 | Light brown | 3 | None | Silt (D), clay (O) | |
| Ven_rep5 | Ven | HSD 31S | 1/4/19 | 55.5114 | 12.4017 | 18.4 | 23 | Light brown | 3 | None | Silt (D), clay (O) | |
| Ven_rep6 | Ven | HSD 31S | 1/4/19 | 55.5127 | 12.4014 | 17.0 | 23 | Light brown | 3 | None | Silt (D), clay (O) | |

| **Table S2:** Overview of species identified by eDNA and morphological inspection. Taxonomic levels are shown for phylum, class, order, family and species. The total number of reads after rarefaction are shown for each species identified by eDNA. The total wet weight for each taxon identified by morphological inspection is shown along with the number of individuals found. Each organism is here categorized based on lifestyle: 1=meiofauna, 2=benthic macrofauna, 3=pelagic organisms, 4=both pelagic organisms and benthic macrofauna. The last column indicates if the species was missing a genetic barcode for eDNA taxonomic assignment (NA for higher than species-level taxonomy). *Note that there was some uncertainty about *Magelona alleni*/*Glycera unicornis* for this taxonomic identification. **Note that this species had 10 reads overall, but dropped to one after rarefaction. | | | | | | | | | | |
| --- | --- | --- | --- | --- | --- | --- | --- | --- | --- | --- |
| **Phylum** | **Class** | **Order** | **Family** | **Species/taxon** | **Rarefied reads** | **Wet weight (g)** | **Total no. of ind.** | **Category** | **Ident. by** | **Missing species barcode** |
| Arthropoda | Hexanauplia | Calanoida | Temoridae | *Temora longicornis* | 8,931 | NA | NA | 1 | eDNA | - |
| Arthropoda | Hexanauplia | Harpacticoida | Ameiridae | *Proameira* sp. | 156 | NA | NA | 1 | eDNA | NA |
| Arthropoda | Hexanauplia | Harpacticoida | Canthocamptidae | *Heteropsyllus curticaudatus* | 1,001 | NA | NA | 1 | eDNA | - |
| Arthropoda | Hexanauplia | Harpacticoida | Canthocamptidae | *Mesochra pygmaea* | 2,977 | NA | NA | 1 | eDNA | - |
| Arthropoda | Hexanauplia | Harpacticoida | Cletodidae | *Cletodes limicola* | 1,327 | NA | NA | 1 | eDNA | - |
| Arthropoda | Hexanauplia | Harpacticoida | Cletodidae | *Cletodes longicaudatus* | 5,354 | NA | NA | 1 | eDNA | - |
| Arthropoda | Hexanauplia | Harpacticoida | Cletodidae | *Cletodes* sp. | 5,184 | NA | NA | 1 | eDNA | NA |
| Arthropoda | Hexanauplia | Harpacticoida | Cletodidae | *Cletodes tenuipes* | 4,066 | NA | NA | 1 | eDNA | - |
| Arthropoda | Hexanauplia | Harpacticoida | Cletodidae | *Enhydrosoma curticauda* | 2,604 | NA | NA | 1 | eDNA | - |
| Arthropoda | Hexanauplia | Harpacticoida | Cletodidae | *Spinapecruris curvirostre* | 611 | NA | NA | 1 | eDNA | - |
| Arthropoda | Hexanauplia | Harpacticoida | Ectinosomatidae | Ectinosomatidae sp. | 11,755 | NA | NA | 1 | eDNA | NA |
| Arthropoda | Hexanauplia | Harpacticoida | Idyanthidae | *Tachidiella minuta* | 610 | NA | NA | 1 | eDNA | - |
| Arthropoda | Hexanauplia | Harpacticoida | Laophontidae | *Laophonte longicaudata* | 1,024 | NA | NA | 1 | eDNA | - |
| Arthropoda | Hexanauplia | Harpacticoida | Miraciidae | *Beatricella aemula* | 97 | NA | NA | 1 | eDNA | - |
| Arthropoda | Hexanauplia | Harpacticoida | Miraciidae | *Delavalia reflexa* | 299 | NA | NA | 1 | eDNA | - |
| Arthropoda | Hexanauplia | Harpacticoida | Miraciidae | *Haloschizopera pygmaea* | 58,108 | NA | NA | 1 | eDNA | - |
| Arthropoda | Hexanauplia | Harpacticoida | Normanellidae | *Normanella mucronata* | 1,600 | NA | NA | 1 | eDNA | - |
| Arthropoda | Hexanauplia | Harpacticoida | Normanellidae | *Normanella obscura* | 2,617 | NA | NA | 1 | eDNA | - |
| Arthropoda | Hexanauplia | Harpacticoida | Rhizotrichidae | *Rhizothrix curvata* | 8,581 | NA | NA | 1 | eDNA | - |
| Gnathostomulida | NA | Bursovaginoidea | Gnathostomulidae | *Gnathostomula armata* | 7,517 | NA | NA | 1 | eDNA | - |
| Kinorhyncha | Cyclorhagida | Kentrorhagata | Semnoderidae | *Semnoderes armiger* | 20,709 | NA | NA | 1 | eDNA | - |
| Nematoda | Chromadorea | Chromadorida | Chromadoridae | Chromadoridae sp. | 209 | NA | NA | 1 | eDNA | NA |
| Annelida | Clitellata | Haplotaxida | Naididae | *Amphichaeta sannio* | 1,025 | NA | NA | 2 | eDNA | - |
| Annelida | Clitellata | Haplotaxida | Naididae | *Tubificoides amplivasatus* | 1,818 | NA | NA | 2 | eDNA | - |
| Annelida | Clitellata | Haplotaxida | Naididae | *Tubificoides benedii* | 196,301 | NA | NA | 2 | eDNA | - |
| Annelida | Clitellata | Haplotaxida | Naididae | *Tubificoides swirencowi* | 14,532 | NA | NA | 2 | eDNA | - |
| Annelida | Clitellata | NA | NA | Oligochaeta sp. | NA | 0.0003 | 1 | 2 | Morph | NA |
| Annelida | Polychaeta | Capitellida | Capitellidae | *Capitella capitata* | NA | 0.001 | 2 | 2 | Morph | - |
| Annelida | Polychaeta | Capitellida | Capitellidae | *Mediomastus fragilis* | NA | 0.0009 | 2 | 2 | Morph | x |
| Annelida | Polychaeta | Capitellida | Capitellidae | *Notomastus latericeus* | NA | 0.4068 | 8 | 2 | Morph | - |
| Annelida | Polychaeta | Capitellida | Maldanidae | *Maldane sarsi* | NA | 0.0201 | 3 | 2 | Morph | - |
| Annelida | Polychaeta | Capitellida | Maldanidae | *Praxillella affinis* | NA | 0.0581 | 2 | 2 | Morph | - |
| Annelida | Polychaeta | Capitellida | Maldanidae | *Praxillura longissima* | NA | 0.4362 | 2 | 2 | Morph | x |
| Annelida | Polychaeta | Capitellida | Maldanidae | *Rhodine gracilior* | NA | 1.1984 | 43 | 2 | Morph | - |
| Annelida | Polychaeta | Cirratulida | Paraonidae | *Aricidea minuta* | 251,162 | NA | NA | 2 | eDNA | - |
| Annelida | Polychaeta | Cirratulida | Paraonidae | *Levinsenia gracilis* | 5,234 | 0.001 | 2 | 2 | Both | - |
| Annelida | Polychaeta | NA | NA | Polychaeta sp. | 5,196 | 0.001 | 2 | 2 | Both | NA |
| Annelida | Polychaeta | Opheliida | Scalibregmatidae | *Scalibregma inflatum* | 108 | 0.0025 | 2 | 2 | Both | - |
| Annelida | Polychaeta | Orbiniida | Orbiniidae | *Leitoscoloplos pugettensis* | 199 | NA | NA | 2 | eDNA | - |
| Annelida | Polychaeta | Orbiniida | Orbiniidae | *Scoloplos armiger* | 119,759 | 0.2352 | 34 | 2 | Both | - |
| Annelida | Polychaeta | Phyllodocida | Glyceridae | *Glycera alba* | NA | 0.0007 | 1 | 2 | Morph | - |
| Annelida | Polychaeta | Phyllodocida | Glyceridae | *Glycera tesselata* | NA | 0.0014 | 1 | 2 | Morph | - |
| Annelida | Polychaeta | Phyllodocida | Glyceridae | *Glycera unicornis* | NA | 0.0022 | 1 | 2 | Morph | - |
| Annelida | Polychaeta | Phyllodocida | Goniadidae | *Goniada maculata* | NA | 0.1752 | 4 | 2 | Morph | - |
| Annelida | Polychaeta | Phyllodocida | Hesionidae | *Nereimyra punctata* | NA | 0.0076 | 1 | 2 | Morph | - |
| Annelida | Polychaeta | Phyllodocida | Hesionidae | *Oxydromus flexuosus* | NA | 0.0097 | 3 | 2 | Morph | - |
| Annelida | Polychaeta | Phyllodocida | NA | Phyllodocida sp. | 5,817 | NA | NA | 2 | eDNA | NA |
| Annelida | Polychaeta | Phyllodocida | Nephtyidae | *Nephtys hombergii* | NA | 0.7296 | 3 | 2 | Morph | - |
| Annelida | Polychaeta | Phyllodocida | Nereididae | *Alitta virens* | 105 | NA | NA | 2 | eDNA | - |
| Annelida | Polychaeta | Phyllodocida | Pholoidae | *Pholoe baltica* | 829 | 0.027 | 18 | 2 | Both | - |
| Annelida | Polychaeta | Phyllodocida | Phyllodocidae | *Eteone longa* | NA | 0.0021 | 2 | 2 | Morph | - |
| Annelida | Polychaeta | Phyllodocida | Phyllodocidae | *Eumida sanguinea* | NA | 0.0008 | 1 | 2 | Morph | - |
| Annelida | Polychaeta | Phyllodocida | Polynoidae | *Bylgides sarsi* | NA | 0.0007 | 3 | 2 | Morph | x |
| Annelida | Polychaeta | Phyllodocida | Polynoidae | *Enipo kinbergi* | NA | 0.0527 | 1 | 2 | Morph | x |
| Annelida | Polychaeta | Phyllodocida | Polynoidae | *Harmothoe imbricata* | NA | 0.0233 | 1 | 2 | Morph | - |
| Annelida | Polychaeta | Phyllodocida | Polynoidae | *Harmothoe impar* | NA | 0.0007 | 1 | 2 | Morph | x |
| Annelida | Polychaeta | Phyllodocida | Polynoidae | *Harmothoe* sp. | NA | 0.0006 | 1 | 2 | Morph | NA |
| Annelida | Polychaeta | Phyllodocida | Syllidae | *Exogone* sp. | NA | 0.0008 | 6 | 2 | Morph | NA |
| Annelida | Polychaeta | Sabellida | Fabriciidae | *Fabricia stellaris* | NA | 0.0002 | 1 | 2 | Morph | - |
| Annelida | Polychaeta | Sabellida | Oweniidae | *Galathowenia oculata* | NA | 0.0105 | 3 | 2 | Morph | - |
| Annelida | Polychaeta | Spionida | Magelonidae | *Magelona alleni** | 252 | 0.1235 | 7 | 2 | Both | - |
| Annelida | Polychaeta | Spionida | Magelonidae | *Magelona minuta* | 127,774 | 0.0021 | 4 | 2 | Both | - |
| Annelida | Polychaeta | Spionida | Magelonidae | *Magelona mirabilis* | NA | 0.0005 | 1 | 2 | Morph | - |
| Annelida | Polychaeta | Spionida | Spionidae | *Dipolydora coeca* | NA | 0.0008 | 1 | 2 | Morph | x |
| Annelida | Polychaeta | Spionida | Spionidae | *Marenzelleria viridis* | 188 | NA | NA | 2 | eDNA | - |
| Annelida | Polychaeta | Spionida | Spionidae | *Polydora cornuta* | NA | 0.0252 | 6 | 2 | Morph | - |
| Annelida | Polychaeta | Spionida | Spionidae | *Polydora* sp. | 1,682 | NA | NA | 2 | eDNA | NA |
| Annelida | Polychaeta | Spionida | Spionidae | *Prionospio cirrifera* | 19,947 | NA | NA | 2 | eDNA | - |
| Annelida | Polychaeta | Spionida | Spionidae | *Prionospio fallax* | 1,581,847 | 0.021 | 21 | 2 | Both | - |
| Annelida | Polychaeta | Spionida | Spionidae | *Pseudopolydora antennata* | NA | 0.0073 | 1 | 2 | Morph | x |
| Annelida | Polychaeta | Spionida | Spionidae | *Pseudopolydora pulchra* | NA | 0.0129 | 1 | 2 | Morph | x |
| Annelida | Polychaeta | Spionida | Spionidae | *Spio armata* | NA | 0.0009 | 1 | 2 | Morph | x |
| Annelida | Polychaeta | Spionida | Spionidae | *Spio filicornis* | NA | 0.0311 | 5 | 2 | Morph | x |
| Annelida | Polychaeta | Spionida | Spionidae | *Spiophanes kroyeri* | 1,837 | 0.0656 | 4 | 2 | Both | - |
| Annelida | Polychaeta | Spionida | Trochochaetidae | *Trochochaeta multisetosa* | 853 | 0.2793 | 3 | 2 | Both | - |
| Annelida | Polychaeta | Terebellida | Ampharetidae | *Ampharete baltica* | NA | 0.0004 | 1 | 2 | Morph | x |
| Annelida | Polychaeta | Terebellida | Ampharetidae | *Ampharete finmarchica* | NA | 0.1036 | 12 | 2 | Morph | - |
| Annelida | Polychaeta | Terebellida | Ampharetidae | *Anobothrus gracilis* | 14,413 | 0.0589 | 9 | 2 | Both | - |
| Annelida | Polychaeta | Terebellida | Cirratulidae | *Caulleriella* sp. | NA | 0.0006 | 1 | 2 | Morph | NA |
| Annelida | Polychaeta | Terebellida | Cirratulidae | *Chaetozone setosa* | NA | 0.0308 | 23 | 2 | Morph | - |
| Annelida | Polychaeta | Terebellida | Cirratulidae | *Chaetozone* sp. | 237,634 | NA | NA | 2 | eDNA | NA |
| Annelida | Polychaeta | Terebellida | Cirratulidae | Cirratulidae sp. | 61 | NA | NA | 2 | eDNA | NA |
| Annelida | Polychaeta | Terebellida | Cirratulidae | *Dodecaceria concharum* | 24 | NA | NA | 2 | eDNA | - |
| Annelida | Polychaeta | Terebellida | Cirratulidae | *Tharyx* sp. | NA | 0.0006 | 1 | 2 | Morph | NA |
| Annelida | Polychaeta | Terebellida | Flabelligeridae | *Diplocirrus glaucus* | 72 | 0.0886 | 9 | 2 | Both | - |
| Annelida | Polychaeta | Terebellida | NA | Terebellida sp. | 50,714 | NA | NA | 2 | eDNA | NA |
| Annelida | Polychaeta | Terebellida | Pectinariidae | *Amphictene auricoma* | NA | 0.0161 | 1 | 2 | Morph | x |
| Annelida | Polychaeta | Terebellida | Pectinariidae | *Pectinaria belgica* | NA | 0.0006 | 1 | 2 | Morph | x |
| Annelida | Polychaeta | Terebellida | Terebellidae | *Lanice conchilega* | NA | 0.8543 | 2 | 2 | Morph | - |
| Annelida | Polychaeta | Terebellida | Terebellidae | *Lysilla loveni* | NA | 2.6148 | 8 | 2 | Morph | x |
| Annelida | Polychaeta | Terebellida | Terebellidae | *Polycirrus* sp. | NA | 0.0259 | 1 | 2 | Morph | NA |
| Annelida | Polychaeta | Terebellida | Trichobranchidae | *Terebellides* sp. | 305 | NA | NA | 2 | eDNA | NA |
| Annelida | Polychaeta | Terebellida | Trichobranchidae | *Terebellides stroemii* | 220 | 1.1978 | 6 | 2 | Both | - |
| Annelida | Polychaeta | Terebellida | Trichobranchidae | *Trichobranchus roseus* | NA | 0.0198 | 2 | 2 | Morph | x |
| Arthropoda | Arachnida | Trombidiformes | Halacaridae | *Rhombognathides pascens* | 136 | NA | NA | 2 | eDNA | - |
| Arthropoda | Hexanauplia | Cyclopoida | Clausiidae | *Rhodinicola elongata* | NA | 0.0002 | 1 | 2 | Morph | x |
| Arthropoda | Hexanauplia | Sessilia | Balanidae | *Amphibalanus improvisus* | NA | 0.6866 | 73 | 2 | Morph | - |
| Arthropoda | Malacostraca | Amphipoda | Ampeliscidae | *Ampelisca tenuicornis* | NA | 0.0012 | 1 | 2 | Morph | - |
| Arthropoda | Malacostraca | Amphipoda | Aoridae | *Lembos websteri* | NA | 0.0019 | 3 | 2 | Morph | - |
| Arthropoda | Malacostraca | Amphipoda | Corophiidae | *Medicorophium affine* | NA | 0.0005 | 1 | 2 | Morph | x |
| Arthropoda | Malacostraca | Amphipoda | Dulichiidae | *Dyopedos porrectus* | NA | 0.0016 | 1 | 2 | Morph | x |
| Arthropoda | Malacostraca | Cumacea | Diastylidae | *Diastylis tumida* | NA | 0.0012 | 1 | 2 | Morph | x |
| Arthropoda | Malacostraca | Cumacea | Leuconidae | *Eudorella emarginata* | NA | 0.0034 | 1 | 2 | Morph | - |
| Arthropoda | Ostracoda | NA | NA | Ostracoda sp. | 132,751 | NA | NA | 2 | eDNA | NA |
| Bryozoa | Gymnolaemata | Cheilostomatida | Membraniporidae | *Membranipora membranacea* | 333 | NA | NA | 2 | eDNA | - |
| Bryozoa | NA | NA | NA | Bryozoa sp. | NA | 0.0033 | 3 | 2 | Morph | NA |
| Chordata | Ascidiacea | Stolidobranchia | Styelidae | *Styela* sp. | NA | 0.4716 | 2 | 2 | Morph | NA |
| Cnidaria | Anthozoa | Actiniaria | Edwardsiidae | *Edwardsia longicornis*** | 1 | NA | NA | 2 | eDNA | - |
| Cnidaria | Anthozoa | Actiniaria | Edwardsiidae | *Edwardsia* sp. | NA | 0.046 | 5 | 2 | Morph | NA |
| Cnidaria | Anthozoa | Actiniaria | Halcampidae | *Halcampa* sp. | 368 | NA | NA | 2 | eDNA | NA |
| Cnidaria | Anthozoa | Pennatulacea | Pennatulidae | *Pennatula phosphorea* | NA | 0.2085 | 2 | 2 | Morph | - |
| Cnidaria | Anthozoa | Pennatulacea | Virgulariidae | *Virgularia mirabilis* | 111,218 | 0.0131 | 1 | 2 | Both | - |
| Cnidaria | Staurozoa | Stauromedusae | Craterolophidae | *Craterolophus convolvulus* | 13 | NA | NA | 2 | eDNA | - |
| Echinodermata | Echinoidea | Clypeasteroida | Fibulariidae | *Echinocyamus pusillus* | 1,117 | 0.0082 | 4 | 2 | Both | - |
| Echinodermata | Echinoidea | Spatangoida | Loveniidae | *Echinocardium cordatum* | 271 | 0.7546 | 3 | 2 | Both | - |
| Echinodermata | Holothuroidea | Apodida | Synaptidae | *Leptosynapta* sp. | 21 | NA | NA | 2 | eDNA | NA |
| Echinodermata | Holothuroidea | Dendrochirotida | Cucumariidae | *Paraleptopentacta elongata* | 12,775 | NA | NA | 2 | eDNA | - |
| Echinodermata | Holothuroidea | Dendrochirotida | Psolidae | *Psolus phantapus* | 27,041 | 15.793 | 1 | 2 | Both | - |
| Echinodermata | Ophiuroidea | Amphilepidida | Amphiuridae | *Amphiura chiajei* | 1,258 | 0.0083 | 2 | 2 | Both | - |
| Echinodermata | Ophiuroidea | Amphilepidida | Amphiuridae | *Amphiura filiformis* | 22,326 | 7.7065 | 117 | 2 | Both | - |
| Echinodermata | Ophiuroidea | Amphilepidida | Amphiuridae | *Amphiura* sp. | NA | 0.0188 | 3 | 2 | Morph | NA |
| Echinodermata | Ophiuroidea | Amphilepidida | Ophiuridae | *Ophiura albida* | NA | 0.3449 | 4 | 2 | Morph | - |
| Echinodermata | Ophiuroidea | Amphilepidida | Ophiuridae | *Ophiura ophiura* | NA | 0.1607 | 1 | 2 | Morph | - |
| Echinodermata | Ophiuroidea | Amphilepidida | Ophiuridae | *Ophiura* sp. | NA | 0.0048 | 5 | 2 | Morph | NA |
| Foraminifera | Monothalamea | Astrorhizida | Astrorhizidae | *Astrorhiza limicola* | NA | 0.0926 | 6 | 2 | Morph | x |
| Mollusca | Bivalvia | Adapedonta | Pharidae | *Phaxas pellucidus* | NA | 0.1852 | 2 | 2 | Morph | - |
| Mollusca | Bivalvia | Cardiida | Cardiidae | *Cerastoderma edule* | NA | 0.0218 | 1 | 2 | Morph | - |
| Mollusca | Bivalvia | Cardiida | Cardiidae | *Parvicardium pinnulatum* | NA | 0.0262 | 2 | 2 | Morph | - |
| Mollusca | Bivalvia | Cardiida | Semelidae | *Abra nitida* | 237,638 | 0.8653 | 46 | 2 | Both | - |
| Mollusca | Bivalvia | Cardiida | Tellinidae | *Fabulina fabula* | NA | 0.2819 | 11 | 2 | Morph | x |
| Mollusca | Bivalvia | Cardiida | Tellinidae | *Macoma balthica* | NA | 0.0011 | 1 | 2 | Morph | - |
| Mollusca | Bivalvia | Cardiida | Tellinidae | *Macoma calcarea* | 50 | NA | NA | 2 | eDNA | - |
| Mollusca | Bivalvia | Cardiida | Tellinidae | *Macomangulus tenuis* | NA | 0.1995 | 2 | 2 | Morph | x |
| Mollusca | Bivalvia | Carditida | Astartidae | *Astarte sulcata* | NA | 0.0112 | 1 | 2 | Morph | - |
| Mollusca | Bivalvia | Galeommatida | Montacutidae | *Kurtiella bidentata* | 48 | 0.2692 | 158 | 2 | Both | - |
| Mollusca | Bivalvia | Galeommatida | Montacutidae | *Tellimya ferruginosa* | NA | 0.003 | 2 | 2 | Morph | - |
| Mollusca | Bivalvia | Lucinida | Thyasiridae | *Thyasira flexuosa* | 22,907 | 1.2651 | 57 | 2 | Both | - |
| Mollusca | Bivalvia | Myida | Corbulidae | *Varicorbula gibba* | NA | 0.6943 | 20 | 2 | Morph | - |
| Mollusca | Bivalvia | Mytiloida | Mytilidae | *Modiolus modiolus* | NA | 39.1561 | 1 | 2 | Morph | - |
| Mollusca | Bivalvia | Nuculida | Nuculidae | *Ennucula tenuis* | 991 | 0.0684 | 13 | 2 | Both | - |
| Mollusca | Bivalvia | Nuculida | Nuculidae | *Nucula nitidosa* | 240 | 1.2737 | 72 | 2 | Both | - |
| Mollusca | Bivalvia | Nuculida | Nuculidae | *Nucula sulcata* | 695 | NA | NA | 2 | eDNA | - |
| Mollusca | Bivalvia | Pholadomyoida | Thraciidae | *Thracia phaseolina* | NA | 0.3191 | 8 | 2 | Morph | - |
| Mollusca | Bivalvia | Sphaeriida | Sphaeriidae | *Pisidium amnicum* | 285 | NA | NA | 2 | eDNA | - |
| Mollusca | Bivalvia | Venerida | Arcticidae | *Arctica islandica* | NA | 78.4028 | 1 | 2 | Morph | - |
| Mollusca | Bivalvia | Venerida | Veneridae | *Chamelea gallina* | NA | 3.0631 | 1 | 2 | Morph | - |
| Mollusca | Caudofoveata | Chaetodermatida | Chaetodermatidae | *Chaetoderma nitidulum* | 3 | NA | NA | 2 | eDNA | - |
| Mollusca | Gastropoda | Cephalaspidea | Cylichnidae | *Cylichna cylindracea* | NA | 0.0029 | 2 | 2 | Morph | - |
| Mollusca | Gastropoda | Cephalaspidea | Philinidae | *Philine aperta* | NA | 0.0136 | 1 | 2 | Morph | - |
| Mollusca | Gastropoda | Cephalaspidea | Retusidae | *Retusa obtusa* | 679 | NA | NA | 2 | eDNA | - |
| Mollusca | Gastropoda | Cephalaspidea | Retusidae | *Retusa truncatula* | NA | 0.0205 | 2 | 2 | Morph | - |
| Mollusca | Gastropoda | Cephalaspidea | Retusidae | *Retusa umbilicata* | 171 | NA | NA | 2 | eDNA | - |
| Mollusca | Gastropoda | Docoglossa | Lottiidae | *Testudinalia testudinalis* | NA | 0.0564 | 1 | 2 | Morph | - |
| Mollusca | Gastropoda | Littorinimorpha | Hydrobiidae | *Peringia ulvae* | 65,560 | 0.4133 | 90 | 2 | Both | - |
| Mollusca | Gastropoda | Littorinimorpha | Iravadiidae | *Hyala vitrea* | NA | 0.0032 | 1 | 2 | Morph | - |
| Mollusca | Gastropoda | Neotaenioglossa | Turritellidae | *Turritellinella tricarinata* | NA | 1.1564 | 4 | 2 | Morph | - |
| Mollusca | Gastropoda | Nudibranchia | Glaucidae | *Facelina bostoniensis* | 133 | NA | NA | 2 | eDNA | - |
| Nematoda | Chromadorea | Monhysterida | Linhomoeidae | *Terschellingia longicaudata* | 20,309 | NA | NA | 2 | eDNA | - |
| Nematoda | NA | NA | NA | Nematoda sp. | NA | 0.004 | 58 | 2 | Morph | NA |
| Nemertea | NA | NA | NA | Nemertea sp. | NA | 0.0341 | 9 | 2 | Morph | NA |
| Nemertea | Palaeonemertea | Carininidae | Tubulanidae | *Carinina ochracea* | 6,204 | NA | NA | 2 | eDNA | - |
| Nemertea | Palaeonemertea | NA | NA | Palaeonemertea sp. | 1,246 | NA | NA | 2 | eDNA | NA |
| Nemertea | Pilidiophora | Hubrechtellidae | Hubrechtidae | *Hubrechtella dubia* | 2,484 | NA | NA | 2 | eDNA | - |
| Phoronida | NA | NA | Phoronidae | *Phoronis* sp. | NA | 8.4847 | 289 | 2 | Morph | NA |
| Platyhelminthes | Rhabditophora | Macrostomida | Microstomidae | *Microstomum* sp. | 213 | NA | NA | 2 | eDNA | NA |
| Platyhelminthes | Rhabditophora | Rhabdocoela | Cicerinidae | *Zonorhynchus seminascatus* | 1,379 | NA | NA | 2 | eDNA | - |
| Platyhelminthes | Turbellaria | NA | NA | Turbellaria sp. | NA | 0.0066 | 2 | 2 | Morph | NA |
| Porifera | Demospongiae | Clionaida | Clionaidae | *Cliona celata* | 301 | NA | NA | 2 | eDNA | - |
| Porifera | NA | NA | NA | Porifera sp. | NA | 0.0396 | 7 | 2 | Morph | NA |
| Priapulida | Priapulimorpha | Priapulimorphida | Priapulidae | *Priapulus caudatus* | 22,083 | 0.0022 | 1 | 2 | Both | - |
| Xenacoelomorpha | NA | Acoela | Childiidae | *Childia macroposthium* | 25 | NA | NA | 2 | eDNA | - |
| Arthropoda | Branchiopoda | Diplostraca | Podonidae | *Evadne nordmanni* | 311 | NA | NA | 3 | eDNA | - |
| Arthropoda | Branchiopoda | Diplostraca | Podonidae | *Pleopis polyphemoides* | 1,528 | NA | NA | 3 | eDNA | - |
| Arthropoda | Branchiopoda | Diplostraca | Podonidae | *Podon intermedius* | 7 | NA | NA | 3 | eDNA | - |
| Arthropoda | Branchiopoda | Diplostraca | Podonidae | *Podon leuckartii* | 64 | NA | NA | 3 | eDNA | - |
| Arthropoda | Branchiopoda | Diplostraca | Sididae | *Penilia avirostris* | 644 | NA | NA | 3 | eDNA | - |
| Arthropoda | Hexanauplia | Calanoida | Acartiidae | *Acartia longiremis* | 13,363 | NA | NA | 3 | eDNA | - |
| Arthropoda | Hexanauplia | Calanoida | Acartiidae | *Acartia tonsa* | 2,360,195 | NA | NA | 3 | eDNA | - |
| Arthropoda | Hexanauplia | Calanoida | Centropagidae | *Centropages hamatus* | 37,579 | NA | NA | 3 | eDNA | - |
| Arthropoda | Hexanauplia | Calanoida | Clausocalanidae | *Microcalanus pusillus* | 192 | NA | NA | 3 | eDNA | - |
| Arthropoda | Hexanauplia | Calanoida | Paracalanidae | *Paracalanus parvus* | 1,575 | NA | NA | 3 | eDNA | - |
| Cnidaria | Hydrozoa | Anthoathecata | Boreohydridae | *Plotocnide borealis* | 20 | NA | NA | 3 | eDNA | - |
| Cnidaria | Hydrozoa | Anthoathecata | Pandeidae | *Leuckartiara octona* | 183 | NA | NA | 3 | eDNA | - |
| Nematoda | Chromadorea | Rhabditida | Raphidascarididae | *Hysterothylacium aduncum* | 138 | NA | NA | 3 | eDNA | - |
| Arthropoda | Hexanauplia | NA | NA | Copepoda sp. | NA | 0.0002 | 2 | 4 | Morph | NA |
| Arthropoda | Hexanauplia | Sessilia | Balanidae | *Balanus* sp. | 884 | NA | NA | 4 | eDNA | NA |
| Arthropoda | Hexanauplia | Sessilia | Verrucidae | *Verruca stroemia* | 267 | NA | NA | 4 | eDNA | - |
| Cnidaria | Hydrozoa | Anthoathecata | Corymorphidae | *Corymorpha nutans* | 6,796 | NA | NA | 4 | eDNA | - |
| Cnidaria | Hydrozoa | Anthoathecata | Corymorphidae | *Euphysa aurata* | 410 | NA | NA | 4 | eDNA | - |
| Cnidaria | Hydrozoa | Leptothecata | Campanulariidae | *Clytia hemisphaerica* | 198 | NA | NA | 4 | eDNA | - |
| Cnidaria | Hydrozoa | Leptothecata | Eirenidae | *Eutonina indicans* | 3 | NA | NA | 4 | eDNA | - |
| Cnidaria | Hydrozoa | NA | NA | Hydrozoa sp. | NA | 0.001 | 1 | 4 | Morph | NA |
| Cnidaria | Scyphozoa | Semaeostomeae | Cyaneidae | *Cyanea capillata* | 837 | NA | NA | 4 | eDNA | - |
